# Carbon nanotubes' surface chemistry determines their potency as vaccine nanocarriers *in vitro* and *in vivo*

**DOI:** 10.1016/j.jconrel.2016.01.030

**Published:** 2016-03-10

**Authors:** Hatem A.F.M. Hassan, Lesley Smyth, Noelia Rubio, Kulachelvy Ratnasothy, Julie T.-W. Wang, Sukhvinder S. Bansal, Huw D. Summers, Sandra S. Diebold, Giovanna Lombardi, Khuloud T. Al-Jamal

**Affiliations:** a Institute of Pharmaceutical Science, Faculty of Life Sciences & Medicine, King's College London, Franklin-Wilkins Building, London SE1 9NH, United Kingdom; b Immunoregulation Laboratory, MRC Center for Transplantation, King's College London, Guy's Hospital, London SE1 9RT, United Kingdom; c College of Engineering, Swansea University, Bay Campus, Fabian Way, Crymlyn Burrows, Swansea SA1 8EN, United Kingdom; d Division of Immunology, Infection, and Inflammatory Diseases, King's College London, Guy's Hospital, London SE1 9RT, United Kingdom

**Keywords:** Vaccine delivery, Carbon nanotubes, Dendritic cells, Nanomedicine

## Abstract

Carbon nanotubes (CNTs) have shown marked capabilities in enhancing antigen delivery to antigen presenting cells. However, proper understanding of how altering the physical properties of CNTs may influence antigen uptake by antigen presenting cells, such as dendritic cells (DCs), has not been established yet. We hypothesized that altering the physical properties of multi-walled CNTs (MWNTs)-antigen conjugates, *e.g.* length and surface charge, can affect the internalization of MWNT-antigen by DCs, hence the induced immune response potency. For this purpose, pristine MWNTs (*p*-MWNTs) were exposed to various chemical reactions to modify their physical properties then conjugated to ovalbumin (OVA), a model antigen. The yielded MWNTs-OVA conjugates were long MWNT-OVA (~ 386 nm), bearing net positive charge (5.8 mV), or short MWNTs-OVA (~ 122 nm) of increasing negative charges (− 23.4, − 35.8 or − 39 mV). Compared to the short MWNTs-OVA bearing high negative charges, short MWNT-OVA with the lowest negative charge demonstrated better cellular uptake and OVA-specific immune response both *in vitro* and *in vivo*. However, long positively-charged MWNT-OVA showed limited cellular uptake and OVA specific immune response in contrast to short MWNT-OVA displaying the least negative charge. We suggest that reduction in charge negativity of MWNT-antigen conjugate enhances cellular uptake and thus the elicited immune response intensity. Nevertheless, length of MWNT-antigen conjugate might also affect the cellular uptake and immune response potency; highlighting the importance of physical properties as a consideration in designing a MWNT-based vaccine delivery system.

## Graphical abstract

**Figure:**
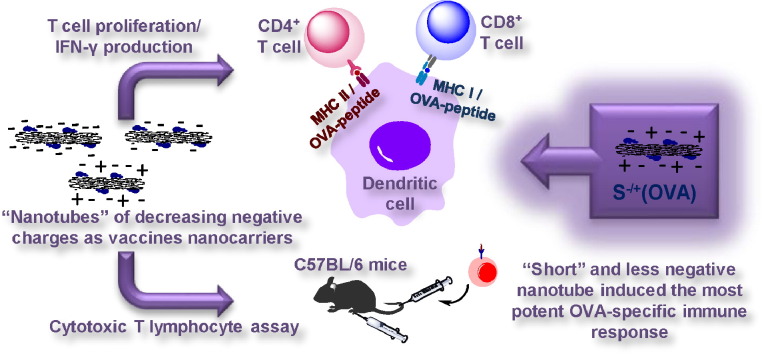

## 1 Introduction

Spherical nanosized vaccine delivery systems, ranging from 15 to 1000 nm, have demonstrated a marked capability in augmenting immune response against the delivered antigens [1], [2], [3]. This has led to clinical investigations of these delivery systems with respect to enhancing the body's immune response against challenging diseases such as cancer [4], [5]. Cylindrical-shaped nanosized delivery systems have also attracted increased interest over the last decades [6]. CNTs are among the most extensively studied cylindrical-shaped delivery systems in the biomedical field [7], [8]. CNTs, owing to high aspect ratio (length to width ratio), have been shown to internalize into cells utilizing both energy-dependent and independent routes. For example, single walled CNT (SWNT) conjugated to fluorescently labeled-DNA or protein was shown to be internalized by HeLa cells *via* energy-dependent endocytosis [9]. Another study reported the internalization of fluorescently-labeled ammonium-functionalized-MWNTs in Jurkat cells, under endocytosis inhibiting conditions, suggesting utilization of energy-independent uptake mechanisms [10].

CNTs have been reported as antigen delivery systems in a number of studies for enhancing the immune response against infectious agents or cancer. In one study, peptide derived from the foot and mouth disease virus (FMDV) was conjugated to SWNT. The binding specificity and biological activity were confirmed using surface plasmon resonance, *in vitro* and *in vivo*, respectively [11], [12], [13]. A stronger immune response, shown by a higher level of anti-FMDV antibodies, was obtained in BALB/C mice immunized with the SWNT-FMDV conjugate compared to the free FMDV [12]. Another study illustrated that conjugation of a malaria-derived peptide to MWNTs induced higher levels of specific antibodies in mice immunized with the conjugate compared to the free peptide [14]. Furthermore, a shift from Th2 to Th1 immune response, marked by increased interferon gamma (IFN-γ) production, was obtained following immunization with SWNT-conjugated tuberculin [15].

Cancer is another disease where CNTs have shown promise as a vaccine delivery tool. Earlier studies explored enhancing delivery of cancer antigens using SWNT or MWNT. Meng et al. reported that immunization with tumor lysate proteins, derived from H22 liver cancer, conjugated to MWNT reduced tumor volume and prolonged the survival of H22 tumor-bearing mice [16]. Conjugation of tumor lysate proteins, derived from MCF7 breast cancer cells, to MWNTs resulted in enhanced DCs uptake and anti-tumor T cell response *in vitro* [17]. Lastly, Villa et al. reported an augmented humoral immune response against a weak immunogenic peptide derived from Wilm's tumor protein, following conjugation to SWNTs [18].

CNT have also been exploited for the delivery of immunoadjuvants such as the synthetic oligodeoxynucleotides containing cytosine-phosphate-guanine motifs (CpG-ODN). Bianco et al. demonstrated improved immune-stimulatory properties of CpG-ODN *in vitro* following non-covalent loading onto cationic SWNTs [19]. Similarly, Zhao et al. reported enhanced cellular uptake of CpG-ODN *in vitro* and *in vivo*. This was associated with an eradication of established intracranially-implanted glioma in mice [20]. In a more sophisticated approach, de Faria et al. utilized MWNTs for the co-delivery of NY-ESO-1 (cancer testis antigen) and CpG-ODN (immunoadjuvant) to DCs *in vivo*. This approach resulted in reduced tumor size and prolonged survival of NY-ESO-1-expressing B16F10-tumor bearing mice challenged with this treatment [21].

All the outlined studies highlighted the immune modulating potential of these cylindrical-shaped nanocarriers and their use as an emerging vaccine delivery system [22], [23]. Despite this, only one study has investigated the relationship between CNT's physical properties, specifically their dimension, and the elicited immune response [24]. In this study, functionalized MWNTs (*f-*MWNTs) of altered physical properties were synthesized to address the structure–activity relationship with respect to influencing antigen presenting cells uptake and immune response *in vitro* and *in vivo*.

## 2 Materials and methods

### 2.1 Mice

All the experiments involving animal use were performed in accordance with the project and personal license authorized by the UK Home Office and the UKCCCR Guidelines (1998). C57BL/6 mice were purchased from Harlan UK (Bicester, UK). OT2 Rag^−/−^ and OT1 Rag^−/−^ mice were maintained at Charles River (Margate, UK). All experiments were carried out using female 6–8 weeks old mice.

### 2.2 Synthesis of functionalized MWNTs (*f-*MWNTs)

**Scheme 1:**
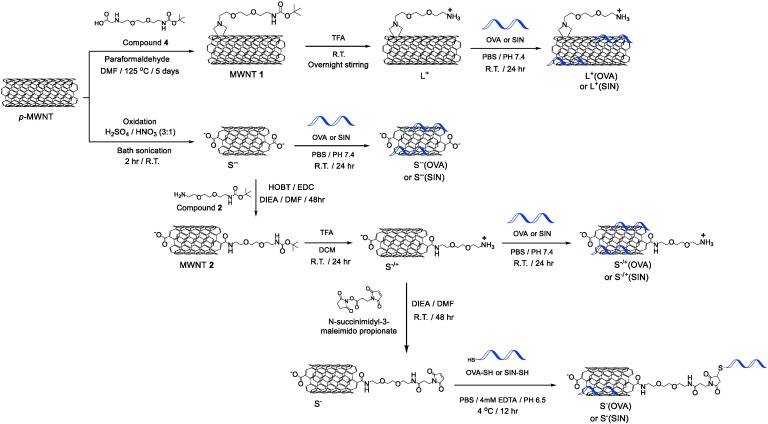
Synthesis of *f-*MWNTs, MWNTs-OVA and MWNTs-SIN. *p*-MWNT was functionalized either *via* 1,3-dipolar cycloaddition or oxidation reactions yielding L^+^ (long *f*-MWNT) or S^−−^ (shortened *f*-MWNT), respectively. S^−−^ was used as a precursor for the synthesis of S^−/+^ that was further functionalized to yield S^−^. Non-covalent conjugation approach was used for OVA loading onto L^+^, S^−−^, or S^−/+^ yielding L^+^(OVA), S^−−^(OVA), or S^−/+^(OVA), respectively. Similarly, non-covalent conjugation was applied for SIN loading onto L^+^, S^−−^, or S^−/+^ yielding L^+^(SIN), S^−−^(SIN), or S^−/+^(SIN), respectively. Covalent conjugation was employed for OVA-SH or SIN-SH loading onto S^−^, yielding S^−^(OVA) or S^−^(SIN), respectively.

The synthetic steps to prepare *f*-MWNTs are described in Scheme 1. Synthetic steps and NMR spectra of compounds **2**, **3** and **4** are illustrated in Scheme S1 and Fig. S1, respectively. Details on the synthesis of *f-*MWNT and the compounds are described in SI.

### 2.3 Synthesis and Characterization of MWNT-OVA or MWNT-SIN conjugates

Details on thiol modification of OVA and solid phase peptide synthesis of SIINFEKL (SIN) or cysteine-modified SIINFEKL (SIN-SH) are described in SI. A solution of 8 mg Albumin chicken egg grade V (OVA, Sigma-Aldrich, UK), or 13 mg SIN in phosphate buffered saline (PBS, Life Technologies, UK) (pH 7.4), was added to a dispersion of 5 mg L^+^, S^−−^ or S^−/+^ in PBS (pH 7.4). The reactions were mixed for 24 h at room temperature. A solution of 8 mg OVA-SH (containing 4 μmol of sulfhydryl group) or 13 mg SIN-SH (containing 10.4 μmol of sulfhydryl group) in PBS (4 mM EDTA, pH 6.5) was added to a dispersion of 5 mg S^−^ (containing 0.4 μmol of maleimide group) in PBS (4 mM EDTA, pH 6.5). The reactions were mixed for 12 h at 4 °C before brief sonication, and filtration through 0.22 μm polycarbonate membrane filters (Merck Millipore, Germany). Solids recovered from MWNTs-OVA or MWNTs-SIN were re-dispersed in appropriate reaction buffer, briefly sonicated and vacuum filtered. Filtrates were collected for the quantification of unreacted OVA or SIN using bicinchoninic acid (BCA) protein assay reagent (Fisher Scientific, UK) as described in SI. The solids recovered from MWNTs-OVA or MWNTs-SIN were re-dispersed in methanol (Fisher Scientific, UK) before being collected by filtration through 0.22 μm polycarbonate membrane filter.

### 2.4 Transmission Electron Microscopy (TEM) and length analysis

The samples of the *f*-MWNTs were dispersed by sonication in de-ionized water at 1 mg/ml, deposited onto a carbon-coated copper TEM grid and dried. Samples were then imaged on a Philips CM 120 Bio-Twin with an accelerating voltage of 120 KV. The lengths of 100 individualized *f-*MWNTs from the TEM images were measured using ImageJ software (National Institute of Health, USA). Results are presented as Box plot graph and the descriptive analysis of length distribution.

### 2.5 Thermogravimetric analysis (TGA)

Weight loss was quantified using a TGA Q500 (TA instrument) with a ramp of 10 °C/min from 100 to 800 °C under nitrogen with a flow rate of 60 ml/min.

### 2.6 Electrophoretic measurements

Zeta potential was determined using a Nanosizer ZS series (Malvern Instruments, Southborough, MA). *f-*MWNTs, MWNT-OVA or MWNTs-SIN were dispersed in 10 times diluted PBS at final *f*-MWNT concentration of 50 μg/ml then transferred to a disposable plain folded capillary Zeta cell [25]. Measurements were carried out at room temperature.

### 2.7 Assessment of *f-*MWNTs or MWNTs-OVA cellular uptake *in vitro*

A 1 mg/ml dispersion of *f-*MWNTs or *f-*MWNTs conjugated to OVA in PBS was prepared. Details on generation of DCs from bone marrow of C57BL/6 are described in SI. Bone marrow derived DCs (BM-DCs) were treated with *f-*MWNTs or MWNTs-OVA each at 10 μg/ml. As a control, BM-DCs were treated with PBS or OVA alone. After 24 h, BM-DCs were harvested, washed with RPMI 1640 medium (Life Technologies, UK) then fixed by the incubation with 4% paraformaldehyde and analyzed using ImageStream 100 cell analyzer (Amins Corporation, USA).

### 2.8 Assessment of the immune response induced by MWNTs-OVA *in vitro* using ^3^H-thymidine incorporation assay

A 0.5 mg/ml dispersion of OVA alone, OVA conjugated to *f-*MWNTs in PBS was prepared. *f-*MWNTs alone were dispersed in PBS at 1 mg/ml. BM-DCs were treated with each of the conjugates at 5 μg/ml OVA. As a control, BM-DCs were treated with PBS or uncoupled *f-*MWNTs. After 24 h, treated BM-DCs were harvested, washed and gamma-irradiated (3000 Gys). CD8^+^ or CD4^+^ T cells were isolated from spleens of OT-I or OT-II mice, respectively, as described in SI. In a 96-well round-bottom plate, CD8^+^ or CD4^+^ T cells were co-cultured with the irradiated BM-DCs at 1:4 in complete medium. The 1:4 ratio was decided from optimization studies (SI). CD8^+^ or CD4^+^ T cells cultured without BM-DCs or with naive BM-DCs were used as controls. Cells were maintained for 3 days/37 °C and the proliferation was measured by adding 1 μCi of ^3^H-thymidine (Perkin Elmer, USA) per well for the last 18 h of culture. Proliferation of CD8^+^ or CD4^+^ T cells was determined by measuring the radiation emitted from the incorporated ^3^H-thymidine using liquid scintillation counter (Wallac 1205 Betaplate) and read as counts per minute (c.p.m.).

### 2.9 Quantification of IFN-γ production using ELISA

IFN-γ present in the culture supernatants collected from BM-DC:T cell co-cultures was determined using anti-mouse IFN-γ sandwich ELISA kit following the manufacturer's protocol (eBioscience). The ELISA plates were measured at 450 nm using FLUOstar Omega, BMG LABTECH (Germany).

### 2.10 Assessment of *f-*MWNTs uptake *in vivo*

C57BL/6 mice (n = 3) were injected *via* the footpad with 100 μg of *f-*MWNTs. Mice were scarified 24 h post injection and the draining popliteal lymph nodes were dissected. The lymph node cells were isolated by incubating the harvested lymph nodes with 50 μl RPMI 1640 medium containing 5 μl of 40 mg/ml collagenase and 2 μl of 0.8 mg/ml DNase (Roche Diagnostics, USA) for 30 min at 37 °C, followed by straining the cells through a 70 μm cell strainer (Becton Dickinson, USA) and washing in PBS (1 ×). Isolated lymph node cells were resuspended in 150 μl PBS and incubated for 30 min at 4 °C with 0.86 μg/ml PE-conjugated mAb against CD11c (PE-CD11c) (Becton Dickinson, USA). Lymph node cells were then washed in PBS and analyzed for side scatter and bright-field intensity using ImageStream 100 cell analyzer (Amins Corporation, USA).

### 2.11 Assessment of MWNTs(DQ-OVA) uptake and antigen processing by DCs *in vivo*

MWNTs(DQ-OVA) were synthesized as described in SI. C57BL/6 mice (n = 3) were injected *via* the footpad with MWNTs(DQ-OVA) each containing 10 μg of DQ-OVA [26]. Mice were scarified 24 h post-injection, the draining popliteal lymph nodes were dissected and cells were isolated and stained with PE-CD11c as described before. Lymph node cells were then analyzed on a FACSCalibur, using CellQuest software (BD Bioscience, CA). Subsequent analysis was done using FlowJo software (TreeStar, Ashland, OR).

### 2.12 Assessment of the immune response induced by the MWNTs-OVA in mice using *in vivo* specific cytotoxic T lymphocyte killing assay

An *in vivo* cytotoxic T lymphocyte (CTL) killing assay was performed using a previously reported method [27]. Briefly, C57BL/6 mice (n = 3) were injected *via* the footpad with PBS, OVA or MWNTs-OVA, each at 50 μg OVA, on days 0, 7 and 14 [28]. On day 21, a 1:1 mixture of 0.5 μM carboxyfluorescein diacetate succinimidyl ester (CFSE, eBioscience, UK)-labeled SIN-pulsed splenocytes and 5 μM CFSE-labeled un-pulsed splenocytes (prepared as described in SI) were administered iv into treated or untreated mice. At 18 h post-injection, mice were scarified; spleens were harvested and splenocytes analyzed using flow cytometric analysis to determine the percentage of SIN-pulsed (0.5 μM CFSE^SIN^) and un-pulsed (5 μM CFSE^no SIN^) cells present. Antigen-specific killing was calculated using the following equation:

$$\left[1–\frac{\left[\text{Percentage}\;\text{of}\;0.5\;\mathrm{\mu M}\;{\text{CFSE}}^{\mathrm{SIN}}\right]}{\left[\text{Percentage}\;\text{of}\;5\;\mathrm{\mu M}\;{\text{CFSE}}^{\mathrm{no}\;\mathrm{SIN}}\right]}\right]\times 100.$$

### 2.13 Statistical analysis

Results are expressed as mean value ± standard deviation (S.D.). Statistical analysis was performed using GraphPad Prism version 5.01, California, USA. Statistical differences were determined using one-way ANOVA followed by Bonferroni post-test.

## 3 Results

### 3.1 Synthesis and characterization of *f-*MWNTs

**Fig. 1:**
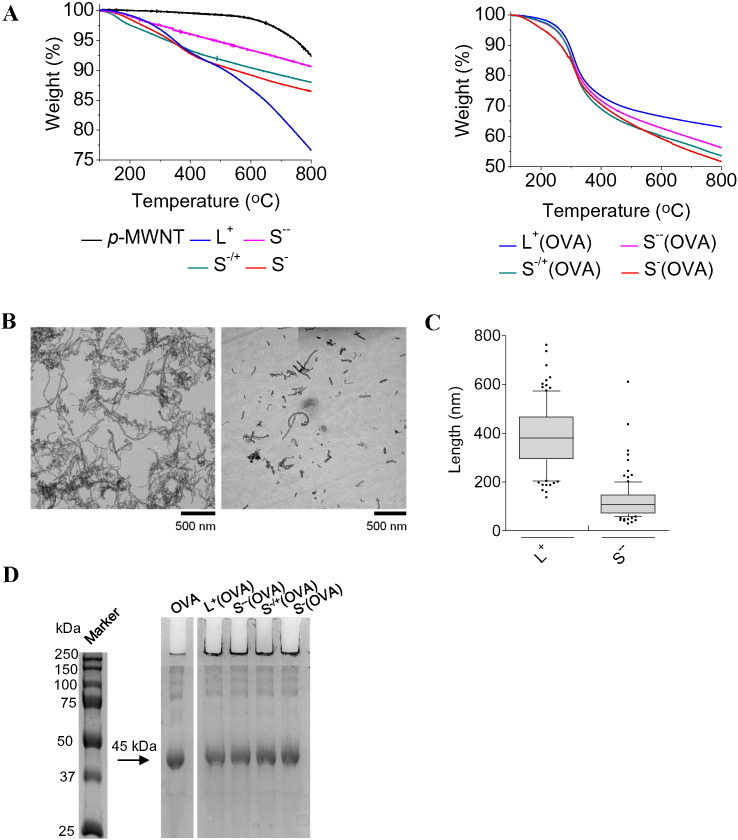
Physicochemical characterization of *f*-MWNTs and MWNTs-OVA. (A) Thermogravimetric profiles of *f-*MWNTs (left) or MWNTs-OVA (right). A known weight of MWNT was exposed to gradually increasing temperature and the weight loss was detected as the temperature increased. *p*-MWNTs were thermally stable up to 600 °C. The weight loss at 600 °C was directly correlated to the amount of introduced functional groups or OVA. Representative thermogravimetric profiles are shown (n = 3). (B) Morphology of *f*-MWNTs. Representative TEM images of L^+^ (left) and S^−−^ (right), deposited on carbon grid from aqueous dispersions. S^−−^ displayed shorter lengths compared to L^+^. (C) Box plot of L^+^ or S^−−^ length distribution. The horizontal line inside the box indicates the median value; the black dots indicate values outside the 10–90 percentiles. Measurements were carried out on 100 individualized nanotubes and analyzed using ImageJ software. (D) Polyacrylamide gel electrophoresis of MWNTs-OVA. MWNTs-OVA were gel electrophoresed using 10% polyacrylamide gel under native gel condition. 10 μg of free OVA or OVA conjugated with MWNTs were loaded in the well. OVA bands were detected by gel staining with brilliant-Coomassie blue. Matching band intensities were observed for both the free OVA and MWNT-conjugated OVA.

The surface of the *p-*MWNTs was chemically modified *via* the incorporation of functional groups to yield *f-*MWNTs (Scheme 1). The first functionalization approach relied on reacting the aromatic rings at the sidewalls of *p-*MWNTs with compound **4** utilizing the previously described 1,3-dipolar cycloaddition reaction [8], [29], [30], yielding MWNT **1**. The Boc group protecting the amine of MWNT **1** was removed using an acidic treatment that yielded L^+^ with positively charged primary amine groups. The second functionalization approach involved shortening *p*-MWNTs by treatment with oxidizing acids that introduce surface defects and negatively charged carboxylic acids yielding S^−−^ [31], [32], [33], [34], [35]. MWNT **2** was synthesized by reacting compound **2** with S^−−^ *via* amide coupling reaction [36], [37]. Boc-deprotection of MWNT **2** liberated the primary amines of S^−/+^. To incorporate functional groups capable of establishing covalent interaction with OVA, S^−/+^ was reacted with a maleimide-terminated spacer, yielding S^−^. Characterization of *f-*MWNTs was achieved using TGA (Figs. 1 A and S2). TGA has shown to be one of the useful techniques to characterize MWNT functionalization [38]. It is based on measuring the weight of the sample being analyzed upon exposing it to a gradually increasing temperature under inert gas (nitrogen). Normally, *p*-MWNTs are thermally stable up to 600 °C above which they dramatically decompose. Functional groups incorporated onto the surface of the *f*-MWNT are, however, less thermally stable and decompose at lower temperatures. The weight loss measured at 600 °C is directly related to the functional groups loading density. The degree of chemical functionalization was calculated using TGA and are summarized in Table 1. Furthermore, the primary amine content of S^−/+^ and S^−^ was qualitatively determined using Kaiser test [39]. The UV–Vis spectra (Fig. S3A) confirmed the reduced primary amine content of S^−^ compared to S^−/+^ as a consequence of the maleimide-terminated spacer addition.

The morphology of an aqueous dispersion of *f-*MWNTs was studied using TEM (Figs. 1B-C and S4). The mean and median lengths of L^+^ were found to be 386 ± 133 nm and 380.6 nm, respectively, whereas S^−−^ exhibited mean and median lengths of 122 ± 82 nm and 107.5 nm, respectively (Table 1, Table 2). Since S^−−^ was the precursor for the synthesis of S^−/+^ and S^−^, the mean length of S^−/+^ and S^−^ was considered to be 122 ± 82 nm. A maximum of one minute sonication steps were applied during washing cycles with organic solvents so that further shortening of *f*-MWNT can be avoided. MWNT-OVA and MWNT-SIN lengths were extrapolated from their *f-*MWNT precursors. Zeta potential of *f-*MWNTs was measured and expressed in Table 1. The fact that S^−/+^ possessed a reduced overall negative charge compared to S^−−^ indicated the presence of residual un-reacted carboxylic acid moieties in S^−/+^. Zeta potential values measured agreed with chemical structures.

### 3.2 Synthesis and characterization of MWNTs-OVA and MWNTs-SIN conjugates

Initially the aim was to conjugate OVA or SIN to *f*-MWNTs, using non-covalent or covalent approaches. Thiol-modification of OVA was achieved using Traut's reagent [40], [41]. SIN and SIN-SH synthesized using solid phase peptide synthesis were characterized using mass spectrometry (Fig. S5). The concentration of the sulfhydryl groups, determined using Ellman's assay [42] was 0.5 μmol or 0.8 μmol per mg of OVA-SH or SIN-SH, respectively (Fig. S3B). As depicted in Scheme 1, L^+^(OVA), S^−−^(OVA) or S^−/+^(OVA) were prepared by non-covalent conjugation of non-modified OVA, while thiol-modified OVA (OVA-SH) was used in preparation of S^−^(OVA) [12], [39]. The same approaches were applied for the conjugation of SIN with *f-*MWNTs yielding L^+^(SIN), S^−−^(SIN), S^−/+^(SIN) or S^−^(SIN). Following their reaction with OVA or SIN, the solids of MWNTs recovered by filtration were analyzed using TGA while the unreacted OVA or SIN contained in the filtrates was quantified using BCA assay. From the thermogravimetric profiles of MWNTs-OVA (Fig. 1A) and the BCA assay, the OVA contents in MWNTs-OVA were calculated and are summarized in Table 1. SIN loading values determined from the thermogravimetric profiles of MWNTs-SIN (Fig. S6) or BCA assay are summarized in Table S1.

TGA showed a mean OVA or SIN loading of 404 or 81 μg per mg *f*-MWNT, respectively. The mean OVA or SIN loading determined using BCA assay was 412 or 78 μg per mg *f*-MWNT, respectively, in agreement with the loading values determined by TGA. Venturelli et al. reported a similar observation on determining the protein loading on CNTs using TGA or by measuring the absorbance of unreacted protein using UV–vis spectroscopy [39]. The surface charges of MWNTs-OVA or MWNTs-SIN are summarized in Tables 1 or S1, respectively.

To further assess OVA and SIN interaction with *f*-MWNTs, MWNTs-OVA and MWNTs-SIN were subjected to native gel electrophoresis (PAGE). OVA contained in MWNTs-OVA exhibited the same migration pattern and band intensity as free OVA (Fig. 1D), suggesting that OVA conjugation with the *f-*MWNTs, even with S^−^, was achieved using non-covalent conjugation. A similar trend was observed for MWNTs-SIN (Fig. S7).

### 3.3 Cellular uptake of *f*-MWNTs by BM-DC does not affect their viability or phenotype *in vitro*

**Fig. 2:**
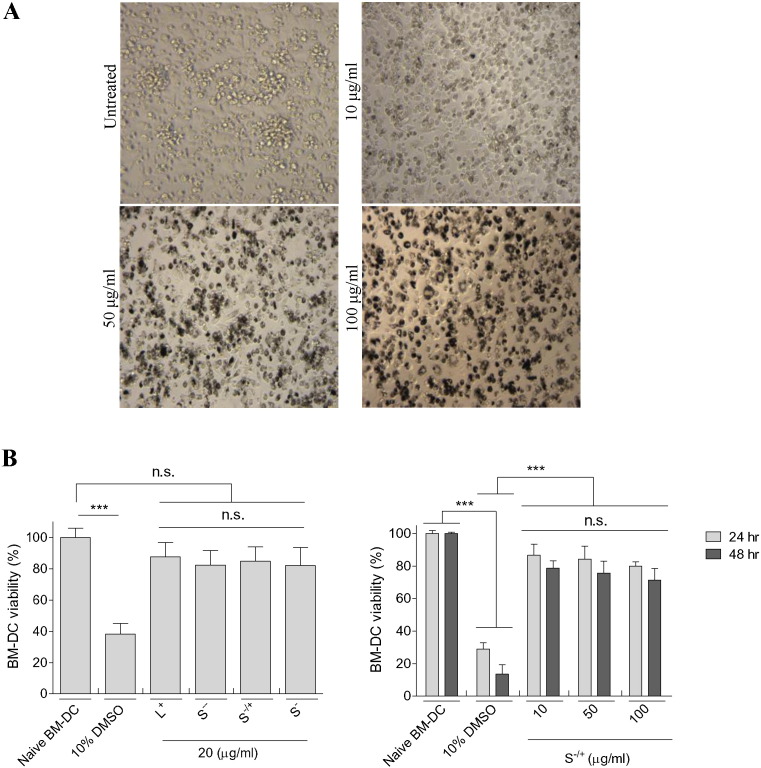
Viability of BM-DCs following treatment with *f-*MWNTs. (A) Light microscopy images of BM-DCs after 24 h incubation with S^−/+^ at 10–100 μg/ml. Images were captured at 20 × magnification. (B) Assessment of *f-*MWNT cytotoxicity using the modified LDH assay. 10% DMSO was used as a positive control. LDH content in the viable BM-DCs was determined in triplicates for each treatment. Results are expressed as mean ± SD (n = 3).

In order to study the effect of *f*-MWNTs on DCs, CD11c^+ ve^ BM-DCs were generated from the bone marrow of C57BL/6 mice (Fig. S8) [43], [44]. First, whether these cells were able to uptake *f*-MWNTs and the effect on BM-DC viability was assessed prior to undertaking further studies. Light microscopy images of *f*-MWNT treated BM-DC revealed the association of the BM-DCs with dark aggregates of *f-*MWNT; however it was difficult to distinguish between cellular uptake of *f-*MWNTs and aggregation of *f-*MWNTs on the cell surface (Fig. 2A). To further assess the cellular uptake of the *f-*MWNTs, BM-DCs were treated with 10 μg/ml of *f*-MWNTs for 24 h before ImageStream analysis.

To overcome the need for fluorescent probes, ImageStream analysis have been previously used to quantify cellular uptake of CNTs for an individual cell, utilizing the CNT ability to absorb and scatter light [45], [46], [47].

**Fig. 3:**
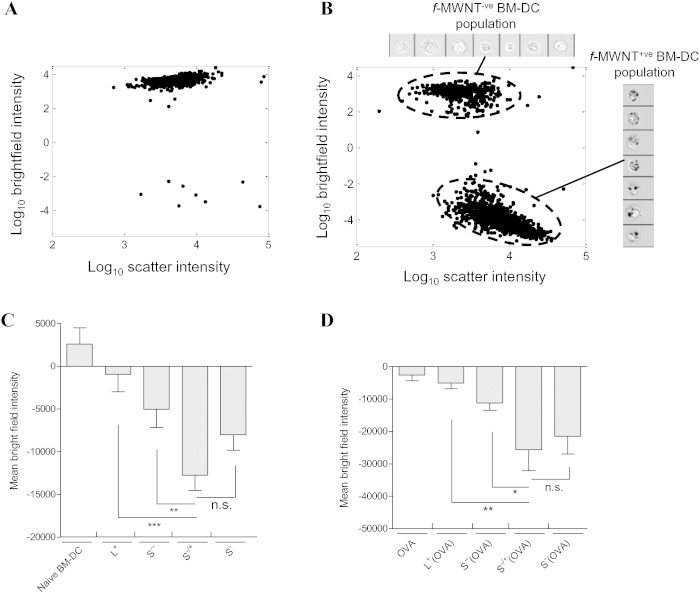
Intracellular uptake of *f-*MWNTs or MWNTs-OVA *in vitro*. BM-DCs were incubated with *f*-MWNTs or MWNTs-OVA each at MWNT concentration of 10 μg/ml for 24 h then analyzed with image stream analysis. (A) Scatter plot of naive BM-DCs. (B) Scatter plot of BM-DCs incubated with S^−/+^, as a representative plot for *f*-MWNT treated BM-DC, showing the S^−/+^ positive and S^−/+^ negative BM-DC populations, identified from mean image intensity in the bright field and scatter channels (cell images are shown in an inset). (C) Relative *f*-MWNTs uptake indirectly determined by measuring the bright field intensity of BM-DCs following treatment with *f-*MWNT. Naive BM-DCs were used as a control. (D) Relative MWNTs-OVA uptake indirectly determined by measuring the bright field intensity of BM-DCs following treatment with MWNTs-OVA. Soluble OVA treated BM-DCs were used as a control. Results are expressed as mean ± SD (n = 3).

As illustrated in Fig. 3A, the scatter plot of naïve BM-DCs appeared as main single population. Following the internalization of *f*-MWNTs, and the associated light scattering, two populations of BM-DCs, namely *f*-MWNT positive (*f*-MWNT^+ ve^) or negative (*f*-MWNT^-ve^) BM-DC population were observed (Figs. 3B and S9). In contrast to the *f*-MWNT^-ve^ BM-DC population, the *f*-MWNT^+ ve^ BM-DC population had light-absorptive black spots of internalized *f-*MWNTs (Fig. 3B). Employing the direct correlation between the reduction in the bright-field intensity and the increase in cellular uptake of CNTs [45], [46], the mean bright-field intensity of BM-DCs treated with the various *f*-MWNTs was measured to quantify *f*-MWNT internalization (Fig. 3C). ImageStream analysis showed that S^−/+^ was significantly acquired by BM-DCs in comparison to L^+^ or S^−−^, while the difference in uptake between S^−/+^ and S^−^ was not significant. Interestingly, positively charged long *f-*MWNT (L^+^) showed the least uptake. The same trend was obtained for *f*-MWNT conjugated to OVA with the highest uptake efficiency being attributed to S^−/+^(OVA) (Fig. 3D).

Acquisition of these molecules was not associated with significant loss of BM-DC viability, as determined by the modified LDH assay [48], even following incubation of these cells with 10–100 μg/ml S^−/+^ for up to 48 h (Fig. 2B).

Materials used in formulating particulate delivery system might have an impact on the induced immune response. For instance, it has previously been reported that DC treatment with poly(lactic-co-glycolic acid) (PLGA) film or PLGA microparticles increased the expression of CD40, CD80 and CD86 [49]. To evaluate whether treatment with *f-*MWNTs or MWNTs-OVA for 24 h affected these co-stimulatory molecules as well as major histocompatibility complex (MHC) levels, BM-DCs were assessed by flow cytometry following incubation with antibodies specific to MHC class I, MHC class II, CD40, CD80 or CD86 (Fig. S10). No significant difference in expression of any of these molecules was detected following *f-*MWNTs-treatment compared to untreated cells. Furthermore, no significant differences were observed following incubation with MWNTs-OVA or OVA.

In conclusion, S^−/+^ and S^−/+^(OVA) treatment of BM-DCs resulted in a higher uptake efficiency compared to *f-*MWNTs and MWNTs-(OVA), respectively. Exposure to these compounds did not affect BM-DC viability nor maturation of these cells.

### 3.4 MWNTs-OVA and MWNTs-SIN augment T cell specific response *in vitro* with varying intensities

**Fig. 4:**
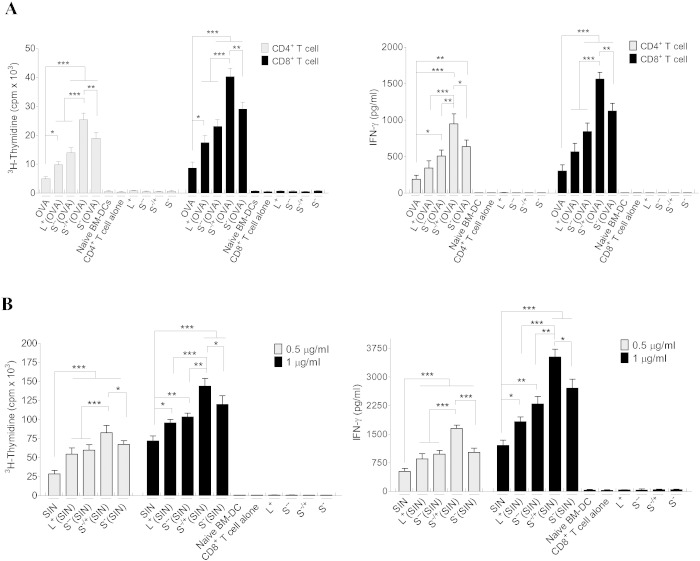
Assessment of the immune response induced *in vitro*. (A, B; left) Determination of T cells proliferation using ^3^H-Thymidine incorporation assay. BM-DCs were incubated with OVA, SIN, MWNTs-OVA, or MWNTs-SIN, each at 5 μg/ml OVA, 0.5 or 1 μg/ml SIN for 24 h. Incubated BM-DCs were harvested, irradiated then co-cultured with CD4^+^ or CD8^+^ T cells at 1:4 ratio for 3 days. CD8^+^ and CD4^+^ T cells proliferation was assessed with ^3^H-thymidine incorporation assay. (A, B; right) Measurement of IFN-γ production in the supernatants of CD4^+^ or CD8^+^ T cells co-cultured for 3 days with OVA, SIN, MWNTs-OVA, or MWNTs-SIN stimulated BM-DCs, by ELISA. Results are expressed as the mean value ± SD (n = 3).

To further assess the efficiency of *f*-MWNTs, to deliver OVA or the MHC I-restricted OVA peptide epitope (SIN) to BM-DCs, we measured their ability to activate antigen specific T cell proliferation and cytokine production. CD8^+^ or CD4^+^ T cells were isolated from the spleens of mice expressing a T cell receptor capable of recognizing OVA peptide SIN (OVA_257–264_) or OVA_323–339_ presented by H-2D^b^ (MHC I) or I-A^b^ (MHC II), respectively (Fig. S11). Initially, we titrated the ratio of SIN pulsed BM-DCs to CD8^+^ T cells and found a DC: T cell ration of 1:4 allowed maximal activation of OVA-specific CD8^+^ T cells (Fig. S12A). In addition, we titrated the concentration of soluble OVA to determine maximum and minimal OVA concentration required to induce T cell stimulation (Fig. S12B). A suboptimal concentration (5 μg/ml) of soluble OVA or OVA contained in MWNTs-OVA was used to determine the differences in T cell activation induced by MWNTs-OVA. BM-DC were cultured with OVA in free form or conjugated with *f-*MWNT and T cell proliferation was assessed by ^3^H-thymidine incorporation. As illustrated in Fig. 4A, MWNTs-OVA treated BM-DCs significantly increased the proliferation of CD8^+^ antigen specific T cells compared to soluble OVA treated BM-DCs. However, significantly higher CD8^+^ T cell proliferation was induced by S^−/+^(OVA) treated compared to L^+^(OVA), S^−−^(OVA) or S^−^(OVA) treated BM-DCs. A similar pattern of proliferation was observed with antigen-specific CD4^+^ T cells. These results show that treatment of BM-DCs with MWNTs-OVA derivatives lead to more efficient antigen presentation compared to antigen in a soluble form and that L^+^(OVA) and S^−/+^(OVA) pulsing induced the least and the highest T cell proliferation rates, respectively. Additionally, IFN-γ production was assessed by ELISA. IFN-γ production correlated with T cell proliferation assay (Fig. 4A).

A dose-dependent CD8^+^ T cell proliferation was obtained on treating the BM-DCs with soluble SIN up to 1 μg/ml (Fig. S12C). As illustrated in Fig. 4B, MWNTs-SIN treated BM-DCs induced significantly stronger CD8^+^ T cell proliferation compared to SIN treated BM-DCs, at concentrations of 0.5 or 1 μg/ml. Furthermore, S^−/+^(SIN) induced a significantly higher CD8^+^ T cell response compared to L^+^(SIN), S^−−^(SIN) or S^−^(SIN). The overall pattern of T cell stimulation and IFN-γ production elicited by MWNTs-SIN was similar to MWNTs-OVA (Figs. 4B and S13).

Although it was demonstrated that the *f-*MWNTs were not capable of affecting the BM-DC phenotypes, further studies were performed to assess the innate immune activation of BM-DCs by *f-*MWNTs. To do this, S^−/+^ was added separately to SIN-pulsed BM-DCs then co-cultured with CD8^+^ T cells. A comparable CD8^+^ T cell proliferation was induced by SIN-pulsed BM-DCs in presence or absence of S^−/+^ (Fig. S14) suggests that *f*-MWNTs lack adjuvanticity and require the antigen to be coupled to them.

Taken together, from these observations we conclude that MWNTs-OVA derivatives were able to induce better CD8^+^ and CD4^+^ T cell responses than soluble OVA, with L^+^(OVA) and S^−/+^(OVA) inducing the least and highest T cell proliferation, respectively, which correlated with the cellular uptake profile. Furthermore and importantly, conjugation of antigen to *f-*MWNT did not appear to interfere with the process of antigen processing.

### 3.5 Cellular uptake of MWNTs-OVA is correlated with the potency of CTL responses induced *in vivo*

**Fig. 5:**
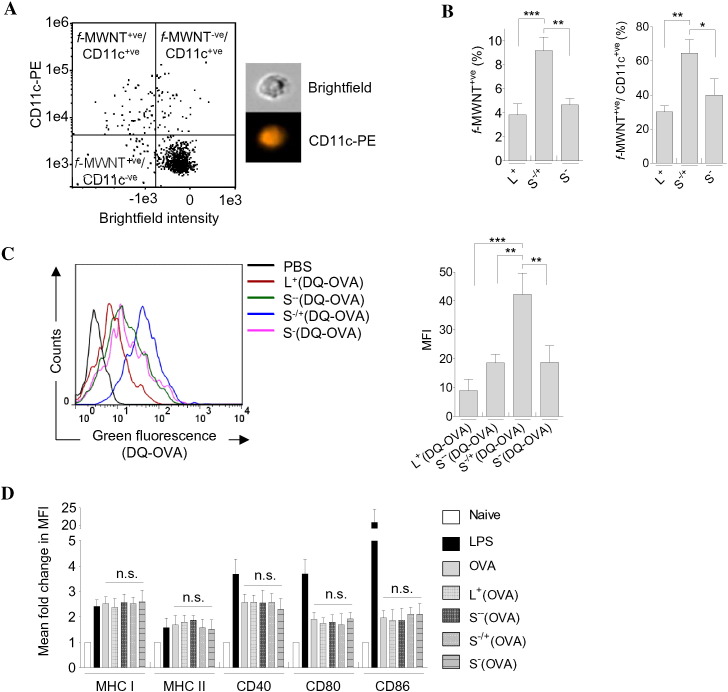
*In vivo* uptake and phenotypic characterization. (A) Uptake of *f-*MWNTs in draining popliteal lymph nodes. C57BL/6 mice (n = 3) were injected *via* the footpad with 100 μg of *f-*MWNTs, and the draining popliteal lymph nodes were dissected 24 h later. The isolated lymph node cells were stained for DCs using PE anti-CD11c (PE-CD11c) and analyzed using ImageStream analysis. Scatter plot of lymph node cells isolated from S^−/+^ injected mouse is shown, as a representative plot, illustrating the gating strategy applied to determine the *f*-MWNT^+ ve^ and CD11c^+ ve^ cells based on the reduction in bright-field intensity and the increase in PE-CD11c fluorescence intensity, respectively (cell images are shown in an inset). (B) Quantification of *f-*MWNTs uptake in popliteal lymph nodes. (Left) Percentage of *f*-MWNT^+ ve^ cells in the whole cell population. (Right) Percentage of *f*-MWNT^+ ve^ cells in the CD11c^+ ve^ cells. (C) Uptake and processing of *f-*MWNT conjugated OVA in draining popliteal lymph nodes. C57BL/6 mice (n = 3) were injected with MWNTs(DQ-OVA), each contained 10 μg DQ-OVA. The isolated lymph node cells were stained with PE-CD11c and analyzed using flow cytometry. (Left) Representative histograms showing the processed DQ-OVA fluorescence, determined using the FL-1 detector. (Right) The MFI of processed DQ-OVA. (D) Effect of MWNTs-OVA on CD11c^+ ve^ lymph node cells phenotypes. C57BL/6 mice (n = 2) were injected with OVA or MWNTs-OVA, each at 50 μg OVA. The isolated lymph node cells were stained with fluorescently-labeled antibodies and analyzed using flow cytometry. The MFI of the positive cells was determined to measure the fold change in the MFI of each marker compared to the naive cells. Results are expressed as mean ± S.D. Statistical analyses were performed using one-way ANOVA with Bonferroni post-test.

To study the cellular uptake of *f-*MWNTs *in vivo*, C57BL/6 mice were injected with *f-*MWNTs and the popliteal lymph nodes were dissected 24 h later. ImageStream analysis revealed that the lymph node cells and the CD11c^+ ve^ lymph node cells internalized S^−/+^ in a significantly higher manner compared to L^+^ or S^−^ [Fig. 5A–B], judging from the bright-field intensity signals. Furthermore, *in vivo* internalization and processing of *f-*MWNT conjugated OVA by the antigen presenting cells was studied by substituting OVA with DQ-OVA in the conjugates. DQ-OVA is a dye-labeled OVA that emits green fluorescence following exposure to proteolytic enzymes [50]. Administration of S^−/+^(DQ-OVA) was associated with highest green fluorescence intensity in the CD11c^+^ lymph node cells [Fig. 5C], determined with flow cytometry analysis. Thus indicating that more DQ-OVA was delivered to CD11c^+^ lymph node cells when conjugated to S^−/+^ than other the other *f-*MWNTs. No significant differences were detected among the phenotypes of the CD11c^+ ve^ lymph node cells derived from MWNTs(OVA) injected mice [Figs. 5D and S15], which was consistent with the *in vitro* phenotypic characterization of BM-DCs. Histological analysis of the main organs excised from injected mice showed no accumulation of MWNTs in lungs, liver, spleen or kidneys (data not shown).

**Fig. 6:**
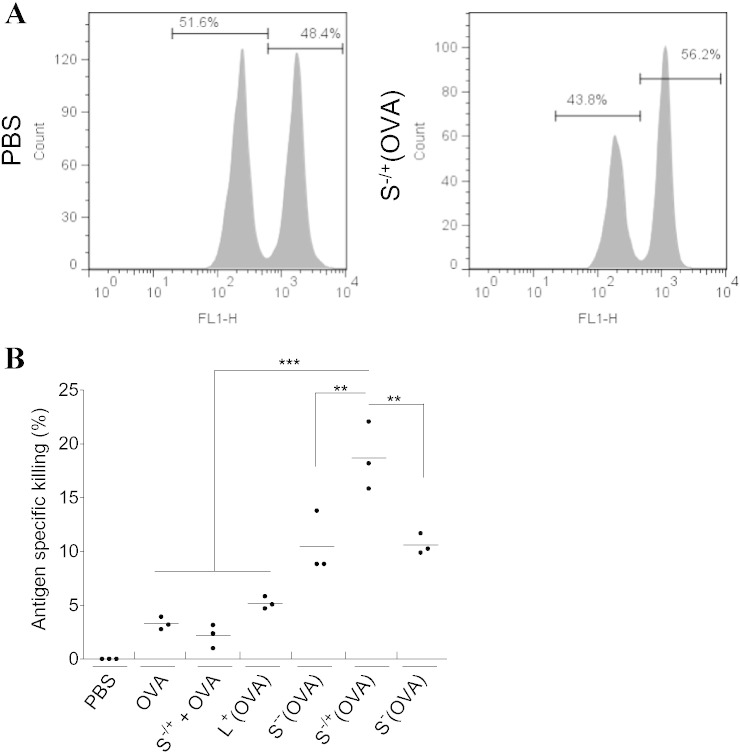
*In vivo* CTL assay. C57BL/6 mice (n = 3) were injected *via* the footpad with OVA or MWNTs-OVA, each at 50 μg OVA on day 0, 7 and 14. Mice injected with PBS were used as a negative control. On day 21 following immunization, target cells derived from naive C57BL/6 mice were intravenously administered to the immunized mice. Splenocytes were harvested from the immunized mice 18 h later and analyzed by flow cytometry to determine the specific killing of the target cells. (A) Representative histograms for splenocytes analyzed by flow cytometry. (B) Specific killing of target cells induced by the different treatments. CTL induced by each individual mouse is represented as a dot, bars represent the mean antigen specific killing for each treatment.

Given the above findings, the *in vivo* efficacy of the MWNTs-OVA conjugates to induce T cell activation *in vivo* was assessed using an *in vivo* CTL assay [27], [28]. C57BL/6 mice were treated with soluble OVA or MWNTs-OVA on days 0, 7 and 14. On day 21 post immunization, mice were injected with a 1:1 splenocyte mixture consisting of 0.5 μM CFSE labeled SIN-pulsed splenocytes (target cells) and 5 μM CFSE labeled un-pulsed splenocytes (control cells). The ratio of the target: control splenocytes was assessed after 24 h using flow cytometry to determine the percentage of antigen specific killing (Fig. 6). Treatment with S^−/+^(OVA) induced the highest percentage of antigen specific killing (18.7% ± 3.1) (P < 0.0001) compared to uncoupled OVA. No significant differences were found between S^−−^(OVA) (10.4% ± 2.86) and S^−^(OVA) (10.6% ± 1.1). Both conjugates, however, showed better efficacy than soluble OVA (3.3% ± 0.6) (P < 0.01) or L^+^(OVA) (5.2% ± 0.58) (P < 0.05). Interestingly, administration of soluble OVA and S^−/+^ did not improve CTL induction compared to treatment with S^−/+^(OVA). It is important to note that overall levels of CTL induction were rather low not exceeding 18.7% of antigen-specific killing. However, this is not surprising taking into account the lack of adjuvanticity of MWNT. In conclusion, consistent with the *in vitro* findings, S^−/+^(OVA) demonstrated the same capabilities on ensuing significantly higher antigen specific immune response than the other MWNTs-OVA *in vivo*.

## 4 Discussion

In this study, we investigated the ability of MWNTs-OVA with different surface functionalities and physical properties to induce antigen-specific immune responses following internalization by the antigen presenting cell. Our *in vitro* studies highlighted differences in T cell activation and cytokine production, for both CD4^+^ and CD8^+^ T cells induced by the different MWNTs-OVA conjugates, which correlated with their cellular internalization. Interestingly, no change in the expression of co-stimulatory molecules was detected among BM-DCs treated with *f-*MWNTs, and the same was observed for MWNTs-OVA treated BM-DCs. This lack of *f*-MWNT's adjuvant properties is in agreement with previously reported studies [17], [19], [51], [52]. These observations might indicate that the pattern of induced immune response was determined by the conjugated antigen uptake by antigen presenting cell and not due to the adjuvanticity of *f*-MWNT. Lastly, cellular uptake and CD8^+^ T cell responses observed *in vivo* were in a good agreement with the *in vitro* studies.

*p*-MWNTs were exposed to various functionalization approaches that yielded long positively charged L^+^ or short negatively charged S^−−^, S^−/+^ or S^−^. OVA conjugation with the *f-*MWNTs yielded MWNTs-OVA of increased negativity. This was in agreement with work reported by de Faria et al. where an increase in the negativity of oxidized MWNT following conjugation with OVA was observed [21]. This could be due to the acidic amino acids content of OVA. Fadel et al. observed the same behavior following the interaction of negatively charged CNTs with streptavidin, neutravidin or avidin bearing net negative, neutral or positive charges, respectively [25]. Taking advantage of their high surface area and surface hydrophobicity, CNTs have been shown to be able to adsorb peptides or proteins of various molecular weights [53]. Based on our findings, we concluded that OVA and its MHC class I-restricted epitope SIN were non-covalently conjugated to *f*-MWNT, possibly *via* π–π stacking or hydrophobic interaction [54].

Bianco et al. assessed the immune response induced by CpG-ODN complexed with either ammonium-functionalized SWNT (ammonium-SWNT) or lysine-functionalized SWNT (lysine-MWNT). The study demonstrated a higher enhancement in immunostimulatory activity of CpG-ODN loaded on the lysine-SWNT than ammonium-SWNT *in vitro* [19]. It was suggested that the more positive lysine-SWNT neutralized the negative charge of CpG-ODN and enhanced its cellular uptake. Li et al. examined the uptake efficiency of 4 types of MWNTs functionalized using 1,3-dipolar cycloaddition (12.95 mV), oxidation (− 52.61 mV), amidation reaction (− 2.35 mV) or polyetherimide-modification (53.33 mV) in BEAS-2B (epithelial cells) and THP-1 (monocytes) cells *in vitro* [55]. A direct correlation between *f-*MWNT's surface positivity and cellular acquisition was found. We have also reported similar findings using a series of cationic dendron-modified MWNT in cancer cells [56], [57]. This was attributed to the enhanced electrostatic interactions between the anionic cell membranes and the cationic *f-*MWNTs.

In case of the shortened *f*-MWNTs series, it is possible that the differences in biological activity, *e.g.*, DCs uptake and immune response are due to reduction in the overall negative charge. One, however, cannot ignore the differences in the chemical structures introduced, which may also have influenced uptake in DC's. Nevertheless, it can be concluded from this study that alteration in *f*-MWNT's surface chemistry may influence the degree of uptake in DC's. The former is directly proportional to the intensity of immune response produced, agreeing with previously reported studies. Previous studies have also demonstrated a correlation between enhanced cellular uptake of positively charged particulate vaccine delivery systems and immune response augmentation. For instance, cationic poly-L-lysine coated nanoparticles (1000 nm in diameter) *in vitro* [58], polystyrene spheres (1000 nm) *in vitro* [59], liposomes (200 nm) of varying lipid composition and surface charges *in vitro* [60], PLGA microspheres loaded with hepatitis B Ag (HBAg) *in vivo* [61], tetanus toxoid-loaded chitosan nanoparticles (40–400 nm) *in vivo* [62] or OVA-conjugated rod-shaped hydrogel nanoparticles *in vitro* and *in vivo* [63].

The positively charged L^+^(OVA) and L^+^(SIN) showed lower immune response intensity compared to the negatively charged S^−/+^(OVA) and S^−/+^(SIN), respectively. These observations might be related to the longer length possessed by L^+^ (~ 386 nm) in contrast to S^−/+^ (~ 122 nm) affecting cellular uptake. The effect of MWNT's length on specific antibody response was previously investigated *in vivo* in New Zealand rabbits and BALB/c mice immunized with protein hapten-MWNTs of two lengths but similar surface charge, and shorter MWNTs (500 nm) induced higher antigen-specific antibody response than the longer MWNTs (> 2 μm) [24].

Size-dependency was also reported for spherical nanoparticles. Previous studies concluded that higher uptake by the antigen presenting cells and a subsequently more potent immune response was induced using smaller sized spherical-shaped particulate vaccine delivery systems [28], [59], [64], [65]. Interestingly, Foged et al. showed that 100 nm nanoparticles, despite being negatively charged, can be taken up more efficiently in DCs than positively charged nanoparticles of bigger size (1000 nm) [59].

It is possible that the differences in immune responses found in our study are due to MWNT's dispersibility characteristics. Short oxidized *f*-MWNTs exhibited enhanced dispersibility and a higher degree of individualization, as observed in the TEM, compared to long non-oxidized *f*-MWNT (L^+^), which displayed a higher tendency of agglomeration. This could be due to the shortening induced by acid-assisted bath sonication [34]. Interestingly, Iannazzo et al. conjugated HIV inhibitor to oxidized-MWNTs or oxidized-MWNTs modified with hydrophilic moieties, and found that increasing the MWNT's dispersibility is associated with higher therapeutic effect of the loaded drug [66]. In our study, the *in vitro* uptake studies demonstrated increased uptake of S^−/+^ or S^−/+^(OVA) on treating the BM-DCs with fixed concentration of *f-*MWNTs or MWNTs contained in MWNTs-OVA, and similar findings were observed *in vivo*. However, variations in antigen loading density among MWNTs-OVA or MWNTs-SIN cannot be ignored among the factors leading to the induced immune response intensities.

Despite the suggestion that differences in immune response efficacy *in vitro* and *in vivo* are related to differences in amounts of nanocarriers and antigen internalized, one cannot exclude the possibility of *f*-MWNT affecting the process of antigen processing by BM-DCs. Exogenous antigens processed in the endocytic compartments of the DCs are loaded onto MHC II molecules and presented to CD4^+^ T cells, while those processed in the cytosolic compartments are loaded onto the MHC I molecules and presented to CD8^+^ T cells. The latter is called cross-presentation [67], [68], [69]. The similar pattern of CD8^+^ T cell stimulation induced by MWNTs-OVA and MWNTs-SIN treated BM-DCs proposes the absence of MWNT's interference with antigen presentation. Thus suggesting that the pattern of induced immune response was dependent on the tendency of MWNTs to enhance the cellular uptake of the conjugated antigen, whether it is an already processed antigen (SIN) or not (OVA).

## 5 Conclusions

Tailoring the physical properties of MWNT-based vaccine delivery systems may increase their efficiency in inducing potent T cell immune responses against challenging infectious or cancer diseases.

## Figures and Tables

**Table 1:** Physicochemical properties of *f*-MWNTs and MWNTs-OVA conjugates.

MWNT	Initial primary amine [final maleimide]^a^^b^^c^ (μmole/g MWNT)	OVA loading (mg/g *f*-MWNT		MWNT length^b^^d^ (nm)	Zeta potential^b^^c^^e^ (mV)
TGA^b^^c^	BCA assay^b^^c^
L^+^	263 ± 72	–	–	386 ± 133	17.3 ± 5.0
L^+^(OVA)	263 ± 72	317 ± 31.1	329 ± 44.0	386 ± 133	5.8 ± 3.2
S^−−^	−	–	–	122 ± 82	− 21.2 ± 3.4
S^−−^(OVA)	−	431 ± 40.0	449 ± 32.5	122 ± 82	− 39.0 ± 4.0
S^−/+^	140 ± 48	–	–	122 ± 82	− 10.1 ± 3.0
S^−/+^(OVA)	140 ± 48	435 ± 28.6	441 ± 36.0	122 ± 82	− 23.4 ± 5.1
S^−^	140 ± 48 [80 ± 25]	–	–	122 ± 82	− 16.4 ± 4.0
S^−^(OVA)	140 ± 48 [80 ± 25]	438 ± 30.5	445 ± 42.2	122 ± 82	− 35.8 ± 3.3

a Analyzed by TGA.

b Data are represented as mean ± SD.

c n = 3.

d Determined from TEM images (n = 100 nanotubes).

e Analyzed by electrophoretic mobility using 10 × diluted PBS buffer.

**Table 2:** Descriptive analysis of L^+^ or S^−−^ length distribution.

*f*-MWNT	Number of nanotubes measured	Minimum (nm)	25% Percentile (nm)	Median (nm)	75% Percentile (nm)	Maximum (nm)	Lower 95% confidence interval of mean (nm)	Upper 95% confidence interval of mean (nm)
L^+^	100	136.131	292.6	380.6	465.9	761.3	359.6	412.8
S^−−^	100	28.607	70.1	107.5	146.9	610.0	106.5	139.3
