# Supplementary material for: Carbon nanotubes' surface chemistry determines their potency as vaccine nanocarriers in vitro and in vivo
Source: J Control Release. 2016 Mar 10;225:205–16. doi: 10.1016/j.jconrel.2016.01.030 (PMC4778609; doi:10.1016/j.jconrel.2016.01.030)
Supplement: Supplementary file 1 — Supplementary material [file mmc1.docx]

Carbon nanotubes' surface chemistry determines their potency as vaccine nanocarriers *in vitro* and *in vivo*

Hatem A. F. M. Hassan^1^, Lesley Smyth^2^, Noelia Rubio^1^, Kulachelvy Ratnasothy^2^, Julie T.-W. Wang^1^, Sukhvinder S Bansal^1^, Huw D. Summers^3^, Sandra Diebold^4^, Giovanna Lombardi^2^, Khuloud T. Al-Jamal^1^ *

Hatem A. F. M. Hassan, Noelia Rubio, Julie T.-W. Wang, Sukhvinder S Bansal, Khuloud T. Al-Jamal

^1^Institute of Pharmaceutical Science, Faculty of Life Sciences & Medicine, King's College London, Franklin-Wilkins Building, London SE1 9NH, United Kingdom

E-mail: [khuloud.al-jamal@kcl.ac.uk](mailto:khuloud.al-jamal@kcl.ac.uk)

Lesley Smyth, Kulachelvy Ratnasothy, Giovanna Lombardi

^2^Immunoregulation Laboratory, MRC Center for Transplantation, King’s College London, Guy’s Hospital, London SE1 9RT, United Kingdom

Huw D. Summers

^3^College of Engineering, Swansea University, Bay Campus, Fabian Way, Crymlyn Burrows, Swansea, SA1 8EN, UK, United Kingdom

# Sandra Diebold

# ^4^Division of Immunology, Infection, and Inflammatory Diseases, King’s College London, Guy’s Hospital, London SE1 9RT, United Kingdom

# *****Corresponding author

### **Supplementary Information**

### **Supplementary materials**

Pristine MWNT (*p*-MWNT), >95% purity, 20-30 nm diameter, 0.5-2 µm length (Stock number: 1237YJS) was obtained from Nanostructured and Amorphous Materials (USA). 2,2'-(ethylene-dioxy)bis(ethylamine), Di-tert-butyl dicarbonate, benzyl bromoacetate, palladium on carbon 10 % W/W, nitric acid 65%, paraformaldehyde, 1-hydroxy-1H-benzotriazole hydrate (HOBt), magnesium sulfate anhydrous, silica gel, potassium permanganate, phenol, pyridine anhydrous, potassium cyanide, N,N-dimethylformamide (DMF), anhydrous 98% , chloroform-D, potassium bicarbonate, piperidine, N,N-diisopropylethylamine (DIPEA), N,N’-diisopropylcarbodiimide (DIC), ethyl 2-cyano-2-(hydroxyimino)acetate (Oxyma), N,N,N’,N’-tetramethylethylenediamine (TEMED), ammonium persulfate, ammonium chloride, ethylenediaminetetraacetic acid (EDTA) and lipopolysaccharide (LPS) from E. coli were obtained from Sigma (UK). Tetrahydrofuran and sulphuric acid 98% were obtained from SLS (UK). Trifluoroacetic acid (TFA), 3-maleimidopropionic acid N-hydroxysuccinimide ester 99% were obtained from Alfa Aesar (UK). Sodium hydroxide, ethyl acetate, hexane fraction from petroleum, diethyl ether, DMF, dichloromethane, dimethyl sulfoxide, Ellman’s reagent and bicinchoninic acid (BCA) protein assay reagent, dialysis tubing MWCO 10000 Da, were obtained from Fisher Scientific (UK) were obtained from Fisher Scientific (UK). DMEM media was obtained from Life Technologies (UK). TLC silica gel 60 F254 25 aluminium sheets, Fluoropore™ Membrane, PTFE, hydrophobic, 0.22 µm, NovaSyn TGR resin, Fmoc-Ser(tBu)-OH, Fmoc-Ile-OH, Fmoc-Asn(Trt)-OH, Fmoc-Phe-OH, Fmoc-Glu(OtBu)-OH, Fmoc-Lys(Boc)-OH, Fmoc-Leu-OH, Fmoc-Cys(Trt)-OH, triisopropylsilane, O-(1 H-6-chlorobenzotriazole-1-yl)-1, 1, 3, 3-tetramethyluronium hexafluorophosphate (HCTU) and trifluoroacetic acid were obtained from Merck Millipore (Germany). Picrylsulfonic acid was purchased from Tokyo Chemical Industry (Japan). Triethylamine, potassium bicarbonate and absolute ethanol absolute were obtained from VWR (USA). 30% Acrylamide/Bis solution and precision plus protein dual xtra standards were obtained from Bio-Rad Laboratories, Inc (USA). RPMI 1640 medium, penicillin, streptomycin, L-glutamine, 2-mercaptoethanol, HEPES buffer solution, Dynabeads® Untouched™ Mouse CD4 Cells kit and DynaMag™-15 were obtained from Life Technologies (UK). 10% heat inactivated foetal calf serum (FCS) was obtained from First Link (UK). Allophycocyanin (APC) hamster anti-mouse CD11c (clone HL3), fluorescein isothiocyanate (FITC) mouse anti-mouse H-2kb (clone AF6-88.5), phycoerythrin (PE) mouse anti-mouse I-A[b (clone AF6-120.1), PE rat anti-mouse CD40 (clone 3/23), PE hamster anti-mouse CD80 (clone 16-10A1), PE rat anti-mouse CD86 (clone GL1), FITC rat anti-mouse CD4 (clone GK1.5), APC rat anti-mouse CD8a (clone 53-6.7), FITC mouse IgG2a, κ isotype control (clone G155-178), PE mouse IgG2a κ isotype control (clone G155-178), PE rat IgG2a κ isotype control (clone R35-95), PE hamster IgG2 κ isotype control (clone B81-3), FITC rat IgG2b κ isotype control (clone A95-1), APC rat IgG2a κ isotype control (clone R35-95) and 70um Cell Strainer were obtained from Becton Dickinson (USA). N-(3-dimethylaminopropyl)-N'-ethylcarbodiimide (EDC) was purchased from AnaSpec Inc. (USA). Ninhydrin was purchased from Santa Cruz Biotechnology (USA). Murine recombinant Granulocyte Macrophage Colony Stimulating Factor (GM-CSF), anti-mouse CD4 [clone YTS 191, rat IgG2b, American Type Culture Collection (ATCC), Manassas, VA], anti-mouse MHC Class II [clone M5/114, rat IgG2b, ATCC], anti-mouse B220 [clone RA3-3A1, rat IgM, ATCC] and anti-mouse CD8 [clone YTS 169, rat IgG2b, ATCC]. Collagenase D and DNase I solution were obtained from Roche Diagnostics (USA).

**Supplementary methods**

**Synthesis of amine and amino acid terminated linkers**

The synthetic scheme is shown in **Scheme S1**.

### Synthesis of {2-[2-(2-amino-ethoxy)-ethoxy]-ethyl}-carbamic acid tert-butyl ester (compound **2**).

To a solution of compound **1**, 2'-(ethylene-dioxy)bis(ethylamine) (15 g, 101 mmol) in tetrahydrofuran (THF) (200 ml), a solution of di-tert-butyl dicarbonate (Boc_2_O) (7 g, 32 mmol) in THF (50 ml) was added, in a dropwise manner at a temperature of 0 ^o^C at a rate of 1 drop/second. The reaction mixture was stirred overnight at room temperature. THF was evaporated under reduced pressure and a white-coloured product was obtained. The reaction crude was suspended in 100 ml ethyl acetate and the unreacted Boc_2_O was removed by washing with 150 ml water followed by extraction of the organic phase. The aqueous phase was washed two more times using ethyl acetate (2 x 100 ml). Organic fractions (300 ml) were collected, dried and evaporated. The final product was purified by silica gel column chromatography. Mobile phase consisting of ethyl acetate/hexane (8:2) was used to elute the di-protected adduct. The polarity of the mobile phase was increased by substituting hexane with methanol to elute the mono-protected product (compound **2**). Fractions containing compound **2** were evaporated and a yellow oily product was obtained and stored at 4 ^o^C.

^1^H NMR (CDCl_3_, ppm) δ: 1.18 (s, 9H), 1.37 (s,2H), 2.62 (t, J = 4.4 Hz, 2H), 3.04 (t, J = 4.4 Hz, 2H), 3.25-3.35 (m, 8H), 5.04 (s, 1H).

### Synthesis of {2-[2-(2-benzyloxycarbonylamino-ethoxy)-ethoxy]-ethyl}-carbamic acid tert-butyl ester (compound **3**).

To a solution of compound **2** (4.88 g, 19.66 mmol) and triethylamine (15.57 ml, 111.71 mmol) in 200 ml THF cooled to 0 ^o^C, a solution of benzyl bromoacetate (1.5 g, 6.55 mmol) in 70 ml THF was added in a dropwise manner at a rate of 1 drop/second. The reaction mixture was stirred overnight at room temperature. THF was evaporated under reduced pressure and the reaction crude was suspended in 100 ml ethyl acetate, washed with 150 ml water and the organic phase was extracted. The aqueous phase was washed two more times using 100 ml of ethyl acetate (2 x 100 ml). Organic fractions (300 ml) were collected, dried and evaporated. The final product was purified with silica column chromatography using ethyl acetate as a mobile phase. Fractions containing compound **3** were evaporated and a yellow oily product was obtained and stored at 4 ^o^C.

**HH_1_**

^1^H NMR (CDCl_3_, ppm) δ: 1.37(s, 9H), 1.97 (s, 1 H), 2.76 (t, J = 4.8 Hz, 2H), 3.25 (s, 2H), 3.44-3.53 (m, 10 H), 5.11 (s, 2H), 5.33 (s, 1 H), 7.4 (s, 5H).

### Synthesis of {2-[2-(2-tert-butoxycarbonylamino-ethoxy]-ethylamino}-acetic acid (compound **4**)

To a solution of compound **3** (1.92 g, 4.84 mmol) in 150 ml methanol, 100 mg of Pd/C (10% w/w) was added. The suspension was placed under H_2_ atmosphere and the reaction mixture was stirred for 4 hr at room temperature. The solution was filtered through a polytetrafluoroethylene (PTFE) filter and the solvent was evaporated under reduced pressure. A yellow oily product was obtained and precipitated several times using methanol/ diethylether until a white precipitate was obtained and stored at 4 ^o^C.

^1^H NMR (CDCl_3_, ppm) δ: 1.43 (s, 9 H), 2.17 (s, 1 H), 3.27 (t, J = 4.4 Hz, 4 H), 3.45-3.64 (m, 8 H), 3.81 (s, 2 H), 5.69 (s, 1 H).

### **Synthesis of f-MWNTs**

### The synthetic scheme is shown in **Scheme 1**.

### Synthesis of MWNT **1**

*p*-MWNT (20 mg) was dispersed in 20 ml of DMF by sonication in a water bath at room temperature for 20 min. A solution consisting of compound **4** (10 mg, 32.6 µmol) and paraformaldehyde (20 mg, 666 µmol) in DMF was added once per day for 5 days. The reaction was performed at 125 ^o^C. After cooling to room temperature, MWNTs were precipitated by the addition of diethylether and filtered through 0.22 µm PTFE membrane. The solid recovered over the filter was dispersed in 100 ml DMF and sonicated in a water bath for 20 min. Unreacted MWNTs were insoluble in DMF and were separated from MWNT **1** by centrifugation at 1000 g. Supernatants were collected and filtered through 0.22 µm PTFE membrane. The solid recovered was washed with diethylether (100 ml), filtered through PTFE membrane, methanol (2 x 100 ml) and filtered through 0.22 µm polycarbonate membrane and dried under vacuum.

### Synthesis of L^+^

MWNT **1** (19 mg) was dispersed in 10 ml TFA and the reaction mixture was stirred overnight at room temperature. The suspension was diluted with dichloromethane then filtered through 0.22 µm PTFE membrane. The resulting solid was washed with methanol (2 x 100 ml) and filtered through 0.22 µm polycarbonate filter. The compound was dialyzed against de-ionized water using a 10,000 Da MWCO dialysis membrane, for 24 hr. The final product was filtered using 0.22 µm polycarbonate filter and dried.

### Synthesis of S^--^

*p*-MWNT (100 mg) was stirred for 2 hr in 24 ml of acid mixture consisting of sulphuric (98 %) / nitric acid (65 %) (3:1 v/v) at room temperature. The reaction mixture was then sonicated for 2 hr in a water bath at room temperature, followed by dilution with 200 ml de-ionized water. Vacuum filtration was performed using 0.22 μm polycarbonate membrane filter. The solid recovered over the filter was washed with methanol, filtered and vacuum-dried.

### Synthesis of MWNT **2**

S^--^ (78 mg, corresponding to 128.3 μmol of COOH content) was dispersed in anhydrous DMF (10 ml). HOBt (89 mg, 657.5 μmol) and EDC (126 mg, 657.3 μmol) were added and the reaction mixture was sonicated for 10 min. Compound **2** (168 mg, 676 μmol) and DIEA (150 μl) were dissolved in 3 ml DMF and added to the reaction mixture. The reaction was stirred for 48 hr at room temperature. The suspension was vacuum filtered using 0.22 μm PTFE membrane filter. The solid recovered over the membrane filter was dispersed in 100 ml DMF, sonicated for 10 min and vacuum-filtered. The solid recovered was washed with methanol (2 x 100 ml), filtered through 0.22 μm polycarbonate membrane filter and dried under vacuum.

### Synthesis of S^-/+^

MWNT **2** (75 mg) was dispersed in 10 ml TFA (10 ml) and stirred overnight at room temperature. The suspension was diluted with dichloromethane then filtered through 0.22 µm PTFE membrane. The resulting solid was washed with methanol (2 x 100 ml) and filtered through 0.22 µm polycarbonate filter. The compound was dialyzed against de-ionized water using a 10,000 Da MWCO dialysis membrane, for 24 hr. The final product was filtered using 0.22 µm polycarbonate filter and vacuum-dried.

### Synthesis of S^-^

S^-/+^ (20 mg, corresponding to 2.8 µmol of amine groups) was dispersed in a solution of 40 µl DIEA in 10 ml DMF. A solution of N-succinimidyl-3-maleimidopropionate (11 mg, 41.3 µmol) in 5 ml DMF was added to the MWNT and the reaction mixture was stirred for 48 hr at room temperature. The suspension was vacuum-filtered through PTFE membrane filter, the solid was recovered, washed with DMF and filtered. Washing was repeated using diethyl ether and methanol and the solid was dried under vacuum.

## **Thiol modification of OVA**

A solution of 2-iminothiolane (14.5 mM, 2 ml) in PBS/ 5 mM EDTA (pH 8) was added to OVA solution (0.6 mM, 1.5 ml) in PBS/ 5 mM EDTA (pH 8). The reaction mixture was mixed for 1 hr at room temperature. Excess 2-iminothiolane was removed by dialysis against PBS buffer (4 mM EDTA, pH 6.5). The thiol groups were quantified using Ellman's assay.

## **Solid phase peptide synthesis of SIINFEKL or SIINFEKLC peptide**

Linear SIINFEKL (SIN) or cysteine-modified derivative (SIN-SH) were synthesized on Fmoc-Rink amide-polyethylene glycol-polystyrene resin (0.41 mmol). Amino acids coupling was performed using a Microwave peptide synthesizer™ (CEM Microwave Technology Ltd) in 2.5 fold excess. Coupling cycles were performed using Fmoc-protected amino acids (1.025 mmol) activated with HCTU (294.76 mg, 0.98 mmol) and DIPEA (255.2 µl, 5 mmol) in 2.5 ml DMF. Deprotection of Fmoc group was performed using 20% piperidine in DMF (v/v). Fmoc Cys(Trt) was activated with DIC (227 uL, 1.025 mmol) and Oxyma (107 uL, 1.025 mmol). Cleavage of the synthesized peptides from the resin was carried out by incubation of 0.65 gm of the resin in a cleavage mixture consisting of 5% phenol, 5% de-ionized water, 90% TFA and 1% triisopropylsilane for 3 hr at room temperature. The resin was filtered and the peptide was precipitated with diethyl ether, followed by centrifugation at 3000 rpm for 10 minutes and the precipitated peptides were collected. The precipitate was washed with diethyl ether twice and then dissolved in de-ionized water and lyophilized. The peptides SIINFEKL-NH_2_ or SIINFEKLC-NH_2_ were characterized by HPLC and with matrix-assisted laser desorption ionization- time of flight mass spectrometry (MALDI-TOF MS) on a Autoflex, Bruker Daltonics instrument using a matrix consisting of 0.3 mg/ml α-cyano-4-hydroxycinnamic acid in a solution of acetonitrile in 0.1% TFA (1:1 v/v).

**Synthesis of MWNTs(DQ-OVA)**

A solution of 1 mg DQ-OVA in 0.5 ml PBS (pH 7.4) was added to a dispersion of 1 mg L^+^, S^--^, S^-/+^ or S^-^ in 1 ml PBS (pH 7.4). The reactions were mixed for 12 hrs at 4 ­^o^C before brief sonication, and filtration through 0.22 μm polycarbonate membrane filters (Merck Millipore, Germany). Solids recovered from MWNTs-(DQ-OVA) were re-dispersed in PBS, briefly sonicated and vacuum filtered. Filtrates were collected for the quantification of unreacted DQ-OVA using bicinchoninic acid (BCA) protein assay reagent.

### **Proton Nuclear Magnetic Resonance (^1^H-NMR)**

^1^H-NMR spectra were recorded in choloroform-d (CDCl_3_ ) using Bruker Avance DRX 400 MHz NMR spectrometer equipped with 5 mm QNP 1H/ 13C probe and fitted with a Z-gradient coil. The peak values are given as ppm (δ), using the residual deuterated solvent protons as reference.

### **UV-Vis Spectroscopy**

UV-Vis spectra were recorded using PerkinElmer, lambda 35 UV/VIS Spectrophotometer between 200 and 800 nm.

### **Chromatography**

The chemical reactions were monitored by thin-layer chromatography (TLC) on silica gel and the products were visualized using an aqueous solution of potassium permanganate consisting of 1 g potassium permanganate, 6.67 g potassium bicarbonate and 1.67 ml 5% sodium hydroxide. Chromatographic purifications were carried out using silica gel packed in a standard column. Organic phases were dried with anhydrous magnesium sulphate.

### **Kaiser Test**

Three solutions were prepared separately; solution I: 1.8 g of phenol in 20 ml ethanol, solution II: 2.2 ml of potassium cyanide 1mM (aqueous solution) dissolved in 98 ml of pyridine, solution III: 1 g of ninhydrin dissolved in 20 ml of ethanol. To 1 mg of MWNT **5** or MWNT **6** the prepared solutions were added in the following order: 125 μl of solution I, 250 μl of solution II and 125 μl of solution III. A blank was prepared by mixing the three solutions using the specified volumes but without the *f*-MWNT. The *f-*MWNTs were sonicated for 3 min in a water bath then heated at 100 ^o^C for 5 min and 4.5 ml of 60% ethanol were added immediately up to 5 ml final volume. The obtained dispersion was centrifuged at 1000 g to precipitate the *f-*MWNTs and the supernatant was collected. A UV/Vis cuvette was filled with the blank to collect the baseline. The absorbance of each sample was detected at 570 nm.

### **Ellman’s assay**

Serial dilutions of cysteine standards ranging from 0.25 to 1.5 mM were prepared using a reaction buffer consisting of 0.1 M sodium phosphate and 1 mM EDTA (pH 8). Ellman’s reagent was dissolved in the reaction buffer at a final concentration of 4 mg/ml. OVA-SH or SIN-SH were dissolved in the reaction buffer at a final concentration of 1 mg/ml. To 2.5 ml of reaction buffer and 50 µl of Ellman’s reagent, 250 µl of each standard, OVA-SH or SIN-SH were added, and then incubated for 15 minutes. The absorbance was measured at 412 nm (Perkin-Elmer Lambda 35 UV vis spectrophotometer). A calibration curve was plotted for the measured absorbance versus cysteine concentration. The sulfhydryl content of OVA-SH or SIN-SH was expressed as µmol per mg OVA-SH or SIN-SH.

### **BCA assay**

The BCA assay was performed following the manufacturer’s instructions with some modifications. The buffer used for sample dilution or standards preparation was PBS (pH7.4) for OVA, DQ-OVA and SIN or 4 mM EDTA in PBS (pH 6.5) for OVA-SH and SIN-SH. A calibration curve was prepared using 25-2000 μg/ml of OVA, OVA-SH, DQ-OVA, SIN or SIN-SH. The formula used to calculate the µg of OVA or SIN per mg of *f*-MWNT was:

$$\frac{\left[ Initial amount of OVA or SIN \left( \mu g \right)- OVA or SIN detected in the filterate \left( \mu g \right) \right]}{\left[ Initial weight of f \mathrm{MWNT}\left( \mathrm{mg} \right) added to the reaction \right]}$$

**Polyacrylamide gel electrophoresis**

The native running 10% gel was prepared using 2.744 ml de-ionized water, 3.3 ml 30% acrylamide, 3.75 ml 1M Tris (pH 8.8), 100 µl 10% ammonium persulfate, 6 µl TEMED. The native running 15% gel was prepared using 1.044 ml de-ionized water, 5 ml 30% acrylamide, 3.75 ml 1M Tris (pH 8.8), 100 µl 10% ammonium persulfate, 6 µl TEMED. Stacking gel was prepared using 3.4 ml de-ionized water, 0.83 ml 30% acrylamide, 0.63 ml 1M Tris (pH 6.8), 50 µl 10% ammonium persulfate, 5 µl TEMED. The loading dye used for the native gel consisted of 1.88 ml 1 M Tris (pH 6.8), 3 ml glycerol and 60 µl 0.03% bromophenol blue which was made up to 10 ml using de-ionized water. To 10 µl of the loading dye, 10 µg of free OVA, SIN or MWNT-conjugated OVA or SIN (equivalent to 10 µg OVA or SIN) were mixed then loaded into the wells of the appropriate gel. Tris-glycine (1X) was used as a running buffer. The bands were visualized by gel staining with comassie blue. Gel imaging was performed using [ChemiDoc™ MP](http://www.bio-rad.com/prd/en/US/LSR/PDP/LOP6DT15/ChemiDoctrade_MP_System) (Biorad, USA).

## **Tissue Culture medium**

The complete culture medium used consisted of RPMI 1640 medium, supplemented with 100 IU/ml penicillin, 100 μg/ml streptomycin, 2 mM L-glutamine, 50 μM 2-mercaptoethanol, 0.01 M HEPES buffer solution and 10% heat inactivated FCS. Cells were incubated in a humidified atmosphere of 5% CO_2_ at 37 ^o^C.

**Generation of DCs from bone marrow**

Bone marrow-derived DCs (BM-DCs) were generated as previously described [[1](#_ENREF_1)]. Femurs and tibiae of female C57BL/6 mice (6-8 weeks) were removed and purified from the surrounding muscle tissues using forceps. Then both ends of the bones were cut with scissors. A 23-gauge needle was filled with 20 ml of the prepared complete medium. The needle was inserted into the bone and the bone marrow was flushed and collected in a Falcon tube followed by washing in complete RPMI medium. Lysis of erythrocytes was done using 5 ml ammonium chloride potassium (ACK) lysis buffer that was prepared using 0.42 g ammonium chloride, 1.8 g EDTA, 50 mg potassium bicarbonate and 50 ml distilled water (pH 7.3-7.4). The ACK lysis buffer was removed by washing the bone marrow-derived cells with complete RPMI medium. Purification of DC precursors was performed by a negative selection process using (DynaMag™-15). Briefly, bone marrow-derived cells were incubated for 30 min at 4 ^o^C with antibodies cocktail consisting of 120 μl of rat mAb reactive against mouse CD4 (YTS 191), MHC Class II (M5/114), B220 (RA3-3A1) or CD8 (YTS 169). Cells were then incubated with superparamagnetic polystyrene beads (Dynabeads^®^) coated with a polyclonal sheep anti-rat IgG Ab. Removal of the undesired cells through immunomagnetic labelling was performed using a magnetic column. After washing, viable cell count was performed by exclusion of 0.5% trypan blue. The bone marrow-derived cells were seeded in a 24 well plate at seeding density of 0.5 x 10 ^6^ cells per well in 1 ml complete medium supplemented with 20 ng/ml GM-CSF (granulocyte-macrophage colony-stimulating factor). On days 3 and 5 of culture, the plate was swirled gently, 800 μl were removed from each well and replaced by fresh 800 μl complete RPMI medium containing GM-CSF. To determine the purity of the BM-DCs, generated cells were stained with APC-conjugated anti-CD11c antibody and analysed using FACS (BD FACSCalibur, CA, USA) operated with Cell Quest™ software (Becton Dickinson, USA) and data analysis was accomplished using FlowJo software (TreeStar Inc., USA). The seeding density of BM-DCs used throughout the *in vitro* experiments was 0.5 x 10 ^6^ cells per well of a 24 well plate and the BM-DCs were treated on their 6^th^ day of culture with the appropriate treatment before harvesting on the next day (7^th^ day).

**Isolation of CD8^+^ or CD4^+^ T cells**

Spleens were isolated from female OT1 Rag^-/-^ or OT2 Rag^-/-^ mice (6-8 weeks) for CD8^+^ or CD4^+^ T cells isolation, respectively. Spleens were passed through a 70 µm cell strainer and splenocytes were collected in RPMI 1640 medium supplemented with 10 μg/ml DNase. Cells were treated with ACK lysis buffer for red blood cell lysis. Further purification of the isolated CD4^+^ T cells by negative selection was done using Dynabeads® Untouched™ Mouse CD4 Cells kit according to the manufacturer’s instructions. Isolated cells were stained with fluorochrome-conjugated antibodies specific against CD8 (CD8^+^ T cell marker) or CD4 (CD4^+^ T cell marker), to determine their purity, and analyzed using FACS (FACSCalibur™, Becton Dickinson, USA) operated with Cell Quest™ software (Becton Dickinson, USA) and data analysis was accomplished using FlowJo software (TreeStar Inc., USA).

## **Assessment of BM-DC viability following treatment with *f-*MWNTs *in vitro***

Modified LDH assay was performed as previously described [[2](#_ENREF_2)]. A dispersion of *f*-MWNT in RPMI 1640 was prepared at 1 mg/ml. BM-DCs were treated with *f*-MWNT at 10-100 μg/ml for 24 or 48 hr. BM-DCs treated with 10% DMSO were used as a positive control. The culture media containing the BM-DCs were collected and centrifuged at 240 x g for 5 min at 4 ^o^C. The supernatant containing the LDH released from the dead cells was discarded. The cell pellets were kept and re-dispersed in 400 μl per well of DMEM media containing 40 μl 9% Triton X-100, then dispensed in a 24-well plate and incubated for 60 min at 37 ^o^C. The cell lysates containing the released LDH were collected and centrifuged at 16000 x g for 5 min at 4 ^o^C to precipitate the *f-*MWNTs. *f-*MWNTs-free cell lysate (20 μl) were carefully transferred to a 96-well plate and mixed with 20 μl of the substrate mix, then the plate was incubated for 15 min at room temperature. The reaction was stopped by the addition of 20 μl stop solution. The absorbance was measured at 490 nm (FLUOstar Omega, BMG LABTECH, Germany). The cell viability was calculated the following equation:

$$\frac{Absorbance of treated cells at 490 nm}{Absorbance of untreated cells at 490 nm} \times100$$

## **Effect of SIN dose titration on DC-induced CD8^+^ T cell proliferation**

A 0.1 mg/ml dispersion of SIN alone in PBS was prepared. BM-DCs were treated with SIN at 0.1, 0.3, 0.7, 1 or 5 µg/ml. The irradiated BM-DCs were co-cultured with CD8^+^ T cells at 1:4 ratio. CD8^+^ T cell proliferation was determined using ^3^H-thymidine incorporation assay.

**Assessment of the immune response induced by MWNTs-SIN *in vitro* by the determination of CD8^+^ T cell proliferation and quantification of IFN-γ production**

*f-*MWNTs alone were dispersed in PBS at 1 mg/ml. A 0.1 mg/ml dispersion of SIN alone or SIN conjugated to *f-*MWNTs in PBS was prepared. BM-DCs were treated with SIN or MWNT-SIN conjugates each at 0.5 or 1 µg/ml SIN. As a control, BM-DCs were treated with the equivalent amount of *f*-MWNT (lacking SIN) contained in the corresponding MWNT-SIN conjugate (10 - 19 μg/ml). The irradiated BM-DCs were co-cultured with CD8^+^ T cells at 1:4 or 1:8 ratio. CD8^+^ T cell proliferation and IFN-γ were determined using ^3^H-thymidine incorporation assay and ELISA, respectively.

**Testing the adjuvant properties of *f*-MWNTs *in vitro***

*f*-MWNT, specifically S^-/+^, was dispersed in PBS at 1 mg/ml. A 0.1 mg/ml dispersion of SIN alone or SIN conjugated to S^-/+^ in PBS was prepared. BM-DCs were treated with free SIN, SIN with S^-/+^ (separately added) or S^-/+^(SIN) each at 1 μg/ml SIN. The irradiated BM-DCs were co-cultured with CD8^+^ T cells at 1:4 ratio. CD8^+^ T cell proliferation was determined using ^3^H-thymidine incorporation assay.

**Determination of BM-DC phenotypes following treatment with *f*-MWNTs or MWNTs-OVA *in vitro***

BM-DCs were treated with OVA, MWNTs-OVA, each at 5 μg/ml OVA, or 15 µg/ml *f-*MWNTs. As a positive control for the induction of BM-DC maturation, 100 ng/ml LPS was used, while untreated BM-DCs were included as a negative control. BM-DCs cultured in the presence of the different conditions were then incubated for 24 hr. Afterwards, BM-DCs were harvested, washed using PBS then dispersed in PBS at 1 x 10^5^ cells per ml. In a 96-well plate, 100 μl of PBS containing 1 x 10^5^ BM-DCs were transferred per well and incubated for 30 min at 4 ^o^C with a mixture of fluorochrome-conjugated mAb. The mixture of mAbs included: APC-conjugated mAb against CD11c (0.86 µg/ml) (BM-DCs are positive for CD11c) and one FITC-conjugated mAb against H-2Kb MHC I alloantigen at 4.3 µg/ml or one PE-conjugated mAb at 1.7 µg/ml specific against one of the following: I-A[b] MHC II alloantigen, CD40, CD80 or CD86. Specific binding of the mAb to the surface antigen was confirmed by incubating the BM-DCs with the relevant isotype control mAb. Isotype mAb used include 4.3 µg/ml FITC conjugated mouse IgG2aκ or 1.7 µg/ml of PE-conjugated mouse IgG2aκ, rat IgG2aκ or hamster IgG2κ. BM-DCs were then washed in PBS (1x) and analyzed on a FACSCalibur, using CellQuest software (BD Bioscience, CA). Subsequent analysis was done using FlowJo software (TreeStar, Ashland, OR). BM-DCs were gated based on the Side Scatter (SSC), Forward Scatter (FSC) and CD11c-APC parameters in order to determine the expression of MHCI, MHC II, CD80, CD86 or CD40 within the BM-DC population only.

**Determination of MWNTs-OVA effects on CD11c^+ve^ lymph node cells phenotypes**

C57BL/6 mice (n=3) were injected *via* the footpad with MWNTs(OVA) each containing 50 µg of OVA. Un-injected mice or mice injected with 10 µg LPS were used as negative or positive controls, respectively. Mice were scarified 24 hrs post injection, the draining popliteal lymph nodes were dissected and lymph node cells were isolated as described before and re-dispersed in 1 ml PBS (1x). In a 96-well plate, 100 μl of PBS containing lymph node cells were transferred per well and incubated for 30 min at 4 ^o^C with a mixture of fluorochrome-conjugated mAb, and analyzed as described above to determine the expression of MHC I, MHC II, CD80, CD86 or CD40 within the CD11c^+ve^ cell population.

**Splenocytes labelling with CFSE**

Naive C57BL/6 mice (n=5) were culled and the spleens were harvested. The splenocytes were isolated by incubating the harvested spleens with 1 ml RPMI containing 25 μl of 40 mg/ml collagenase and 10 μl of 20 mg/ml DNase for 30 minutes at 37 ^o^C, followed by straining the cells through a 70 μm cell strainer. The isolated splenocytes were divided into two equal portions then the cells were spun and resuspended in 5 ml RPMI. One portion was pulsed with 200 nM SIN and subsequently labelled with 0.5 µM CFSE while the other portion was left un-pulsed and labelled with 5 µM CFSE.

### **Supplementary Figures**

**Scheme S1. Synthetic scheme of compounds 2 and 4.**

**Table S1. Physicochemical properties of *f*-MWNTs and MWNTs-SIN conjugates.**

| MWNT | Initial primary amine  [final maleimide]^[a] [b] [c]^  (µmole/ g MWNT) | SIN loading  (mg/ g *f-*MWNT) | | MWNT length^[b] [d]^ (nm) | Zeta potential^[b] [c] [e]^ (mV) |
| --- | --- | --- | --- | --- | --- |
|  |  | TGA^[b] [c]^ | BCA assay^[b] [c]^ |  |  |
| L^+^ | 263 ± 72 | - | - | 386 ± 133 | 17.3 ± 5.0 |
| L^+^(SIN) | 263 ± 72 | 57 ± 10.8 | 52 ± 15.1 | 386 ± 133 | 12.3 ± 3.6 |
| S^--^ | - | - | - | 122 ± 82 | -21.2 ± 3.4 |
| S^--^(SIN) | - | 97 ± 27.6 | 86 ± 32.0 | 122 ± 82 | -25.5 ± 5.1 |
| S^-/+^ | 140 ± 48 | - | - | 122 ± 82 | -10.1 ± 3.0 |
| S^-/+^(SIN) | 140 ± 48 | 88 ± 16.6 | 83 ± 27.8 | 122 ± 82 | -13.1 ± 4.3 |
| S^-^ | 140 ± 48 [80 ± 25] | - | - | 122 ± 82 | -16.4 ± 4.0 |
| S^-^(SIN) | 140 ± 48 [80 ± 25] | 90 ± 21.8 | 93 ± 25.4 | 122 ± 82 | -21.2 ± 3.9 |

^[a]^ Analyzed by TGA.

^[b]^ Data are represented as mean ± SD.

^[c]^ n= 3.

^[d]^ Determined from TEM images (n= 100 nanotubes).

^[e]^ Analyzed by electrophoretic light scattering using 10x diluted PBS buffer.

| MWNT | Initial primary amine  [final maleimide]^[a] [b] [c]^  (µmole/ g MWNT) | SIN loading  (mg/ g *f-*MWNT) | | MWNT length^[b] [d]^ (nm) | Zeta potential^[b] [c] [e]^ (mV) |
| --- | --- | --- | --- | --- | --- |
|  |  | TGA^[b] [c]^ | BCA assay^[b] [c]^ |  |  |
| L^+^ | 263 ± 72 | - | - | 386 ± 133 | 17.3 ± 5.0 |
| L^+^(SIN) | 263 ± 72 | 57 ± 10.8 | 52 ± 15.1 | 386 ± 133 | 12.3 ± 3.6 |
| S^--^ | - | - | - | 122 ± 82 | -21.2 ± 3.4 |
| S^--^(SIN) | - | 97 ± 27.6 | 86 ± 32.0 | 122 ± 82 | -25.5 ± 5.1 |
| S^-/+^ | 140 ± 48 | - | - | 122 ± 82 | -10.1 ± 3.0 |
| S^-/+^(SIN) | 140 ± 48 | 88 ± 16.6 | 83 ± 27.8 | 122 ± 82 | -13.1 ± 4.3 |
| S^-^ | 140 ± 48 [80 ± 25] | - | - | 122 ± 82 | -16.4 ± 4.0 |
| S^-^(SIN) | 140 ± 48 [80 ± 25] | 90 ± 21.8 | 93 ± 25.4 | 122 ± 82 | -21.2 ± 3.9 |

**Table S2. DQ-OVA content of MWNTs(DQ-OVA).**

| MWNT(DQ-OVA) | DQ-OVA content (mg/g *f-*MWNT) |  |
| --- | --- | --- |
| L^+^(DQ-OVA) | 174 | |
| S^--^(DQ-OVA) | 279 | |
| S^-/+^(DQ-OVA) | 251 | |
| S^-^(DQ-OVA) | 257 | |


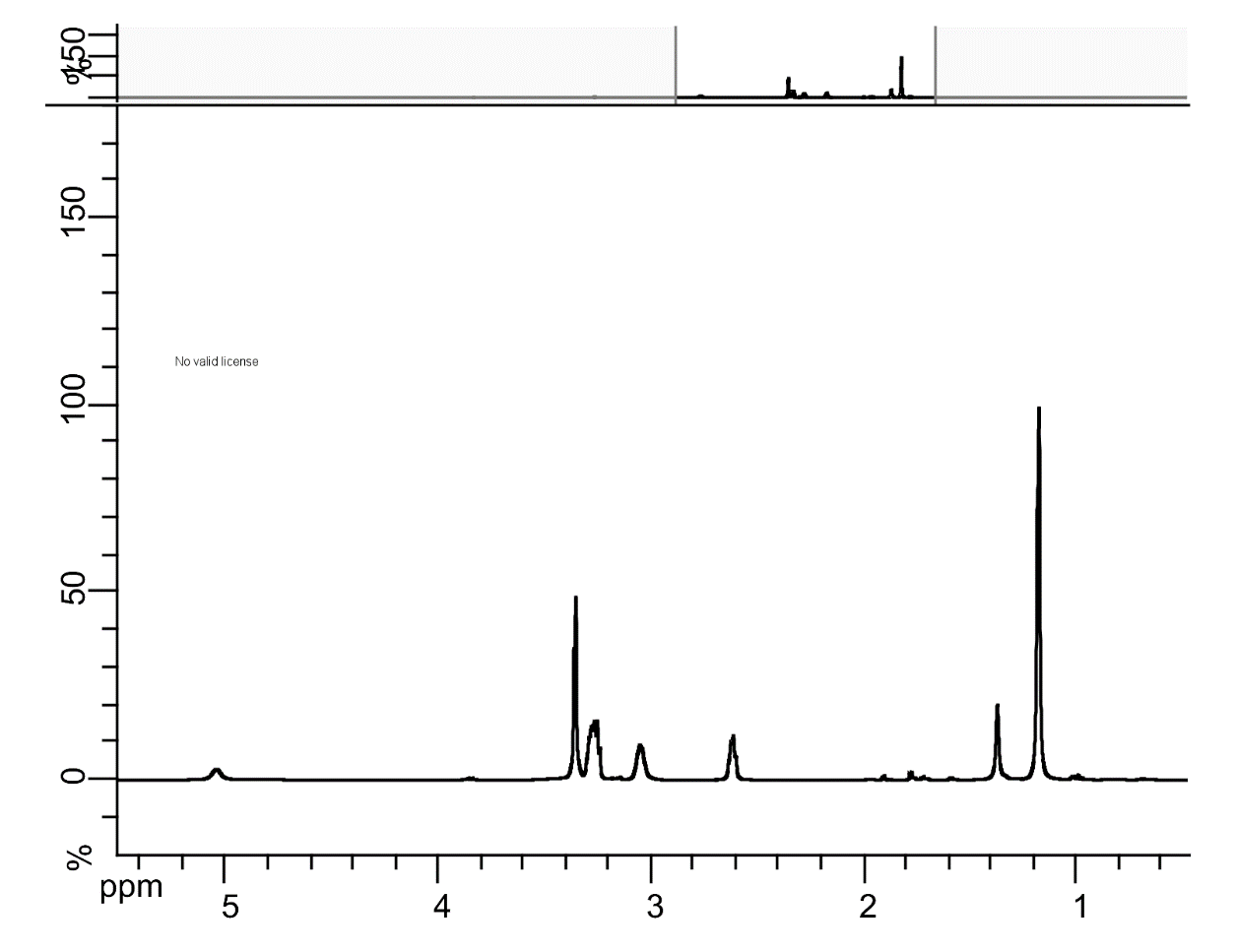

a

a

a

a

a

a

a

a

a

f

c

c

d

d

d

d

d

d

d

d

c

c

b

e

e

e

a

a

a

a

a

a

a

a

a

f

d

d

e

e

e

e

e

e

e

e

c

c

b

b

b

c

d

f

0.7

8.1

2.0

1.8

2.1

9.0

a

a

a

a

a

a

a

a

a

g

c

c

e

e

e

e

e

e

e

e

e

e

d

d

f

f

h

h

h

h

h

**
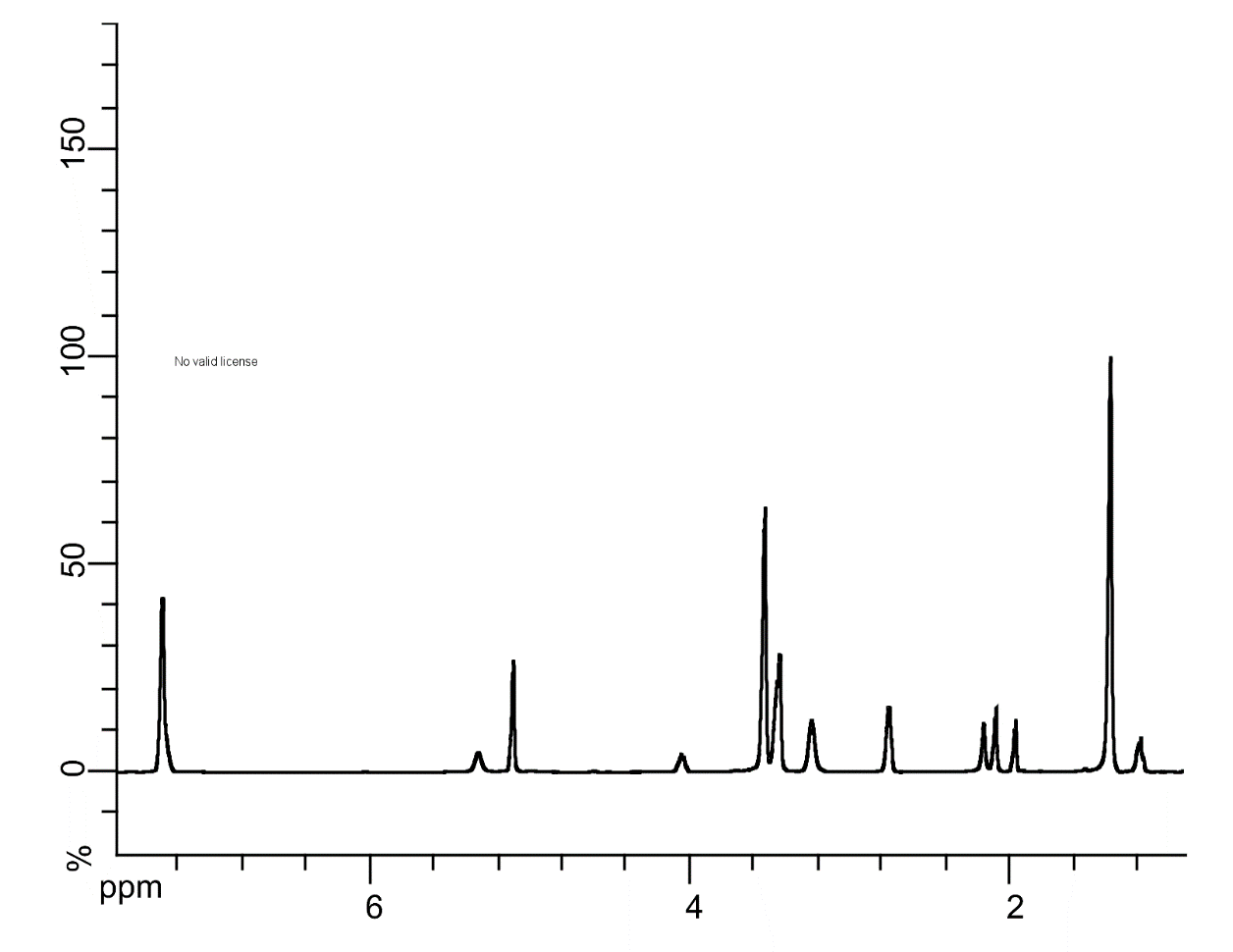
**

e

a

h

f

b

c

d

4.8

0.8

2.0

10.0

2.0

2.0

1.0

9.0

g

**
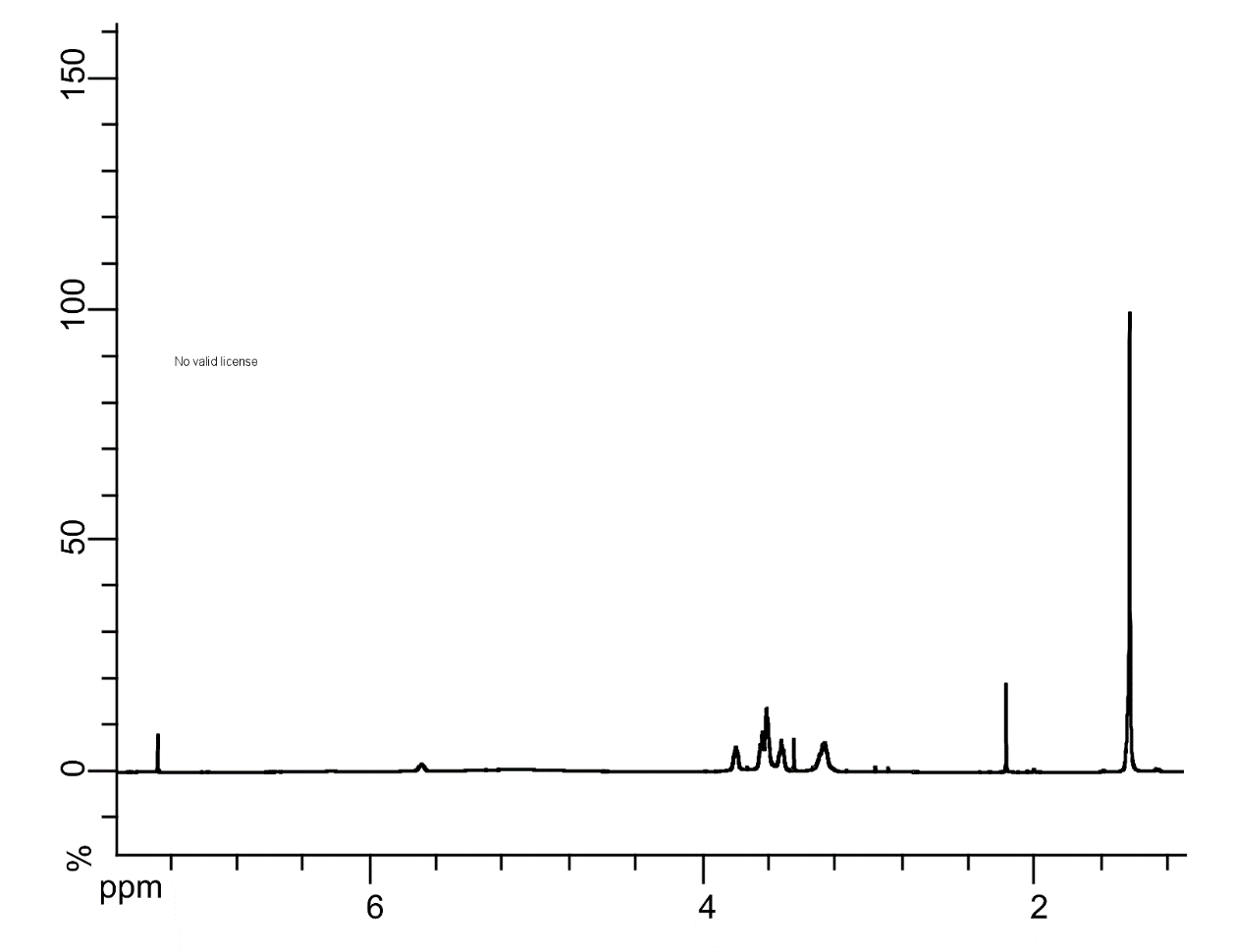
**

a

d

b

e

c

f

0.7

2.0

7.8

3.6

0.6

9.0

**Figure S1. H^1^NMR spectra of compounds 2, 3 and 4.**

**Figure S2. Characterization of *f-*MWNTs by TGA.** A known weight of MWNT was exposed to gradually increasing temperature and the weight loss was detected as temperature increased. *p*-MWNTs were thermally stable up to 600 ^o^C. The weight loss at 600 ^o^C was directly correlated to the amount of introduced functional groups. Representative thermogravimetric profiles are shown (n=3).

**A**

**B**

**Figure S3. Detection of primary amine or sulfhydryl groups using Kaiser test or Ellman’s assays, respectively.** (A) UV-Vis spectra of the chromophoric compound formed between Ninhydrin and primary amines, showing the characteristic absorbance at λ_max_ of 570 nm (S^-/+^) that was reduced following the maleimide group introduction (S^-^). (B) UV-Vis spectra of OVA, OVA-SH, SIN or SIN-SH following the reaction with Ellman’s reagent showing the increase in absorbance at the λ_max_ after modification with sulfhydryl groups using Traut’s reagent or cysteine as expected.

**B**


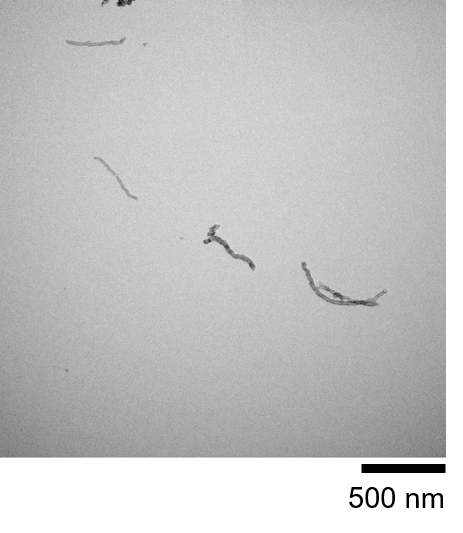


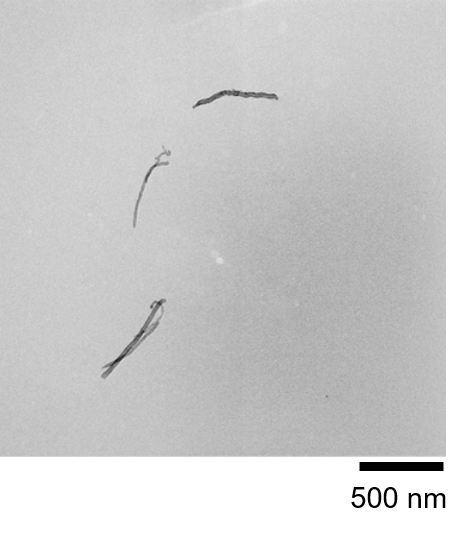


**A**

**
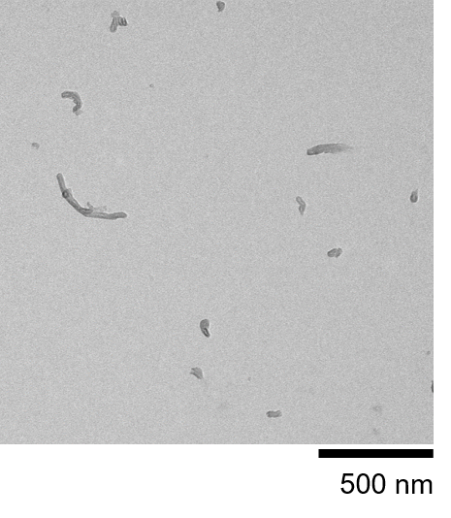
**

**D**

**C**

**
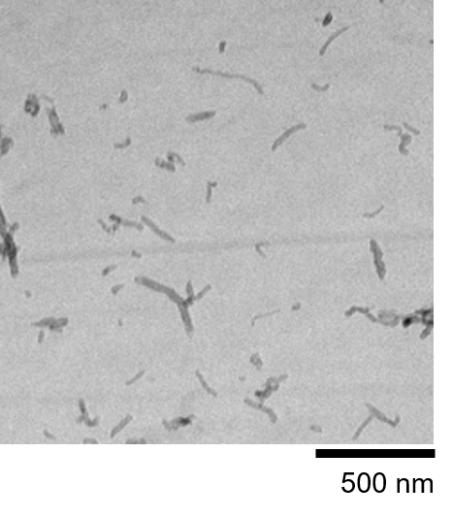
**

**Figure S4. Additional TEM images of L^+^ (A-B) or S^--^ (C-D), dispersed at 1 mg/ml in de-ionized water.**

986 [SIN + Na]^+^

1067 [SIN-SH + H]^+^

1089 [SIN-SH + Na]^+^

**Figure S5. MALDI Mass Spectra of SIN (MW= 963 g/mol) and SIN-SH (MW= 1066.29 g/mol).**

**Figure S6. Thermogravimetric profiles of MWNTs-SIN conjugates.** Representative thermogravimetric profile of 3 runs is shown.


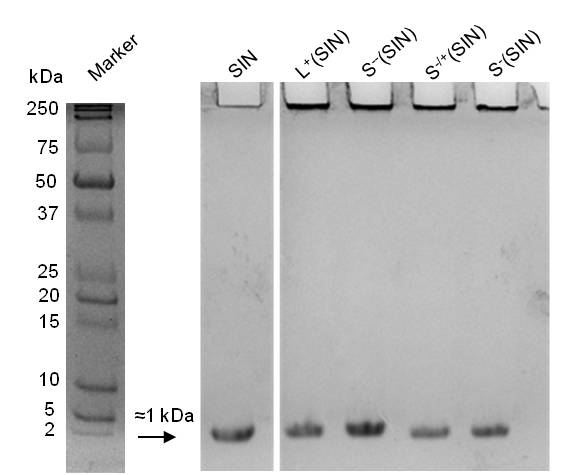


**Figure S7. Polyacrylamide gel electrophoresis of MWNTs-SIN conjugates.** MWNTs-SIN conjugates were gel electrophoresed using 15% polyacrylamide gel under native gel condition. 10 μg of free SIN or SIN conjugated with MWNTs were loaded in the well. SIN bands were detected by staining the gel with brilliant-coomassie blue. Matching band intensities were observed for both free SIN and MWNT-conjugated SIN.

**Figure S8. Characterization of BM-DCs by immunostaining.** The viable BM-DCs were gated (Left) to determine their CD11c marker expression (Right) using flow cytometry.

**Figure S9. Intracellular uptake of *f-*MWNTs *in vitro.*** Scatter plot of BM-DCs incubated with L^+^, S^--^ or S^-^determined using ImageStream analysis.

MHC I

MHC II

CD40

CD80


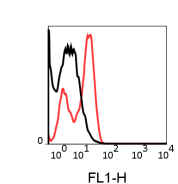


Naive

BM-DC


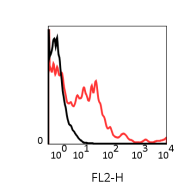

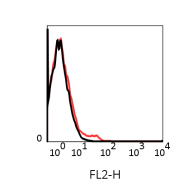

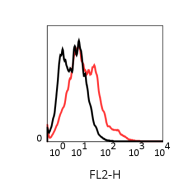

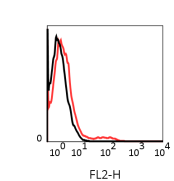


CD86


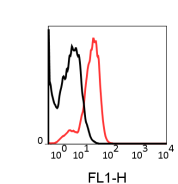


LPS


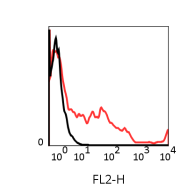

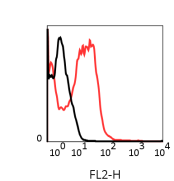

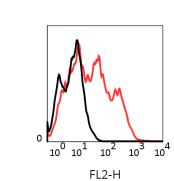

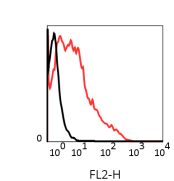

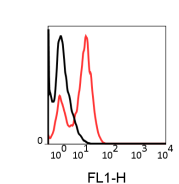


L^+^


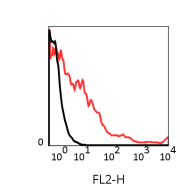

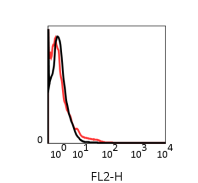

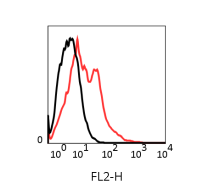

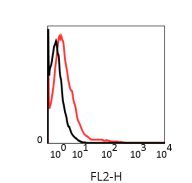

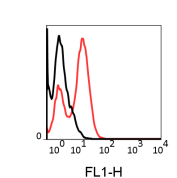


S^--^


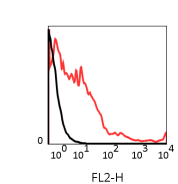

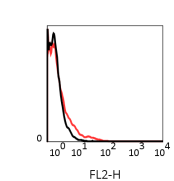

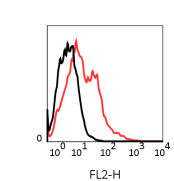

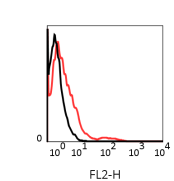

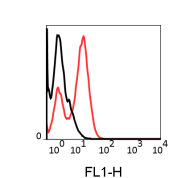


S^-/+^


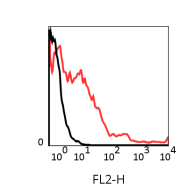

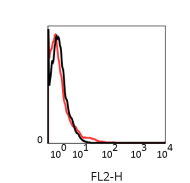

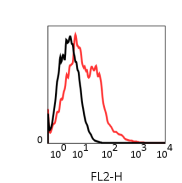

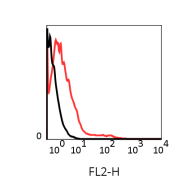

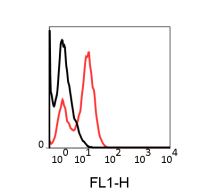


S^-^


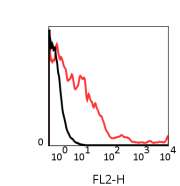

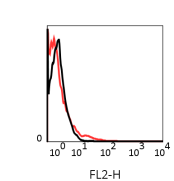

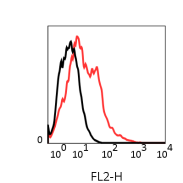

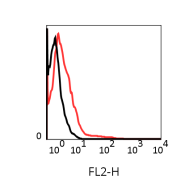

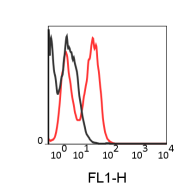


OVA


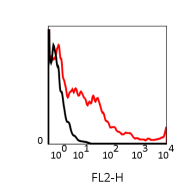

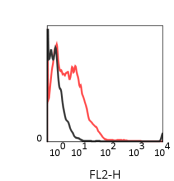

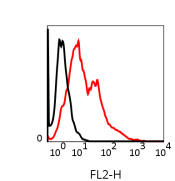

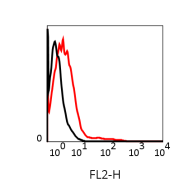

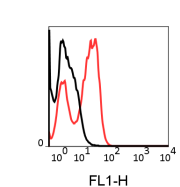


L^+^(OVA)


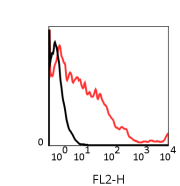

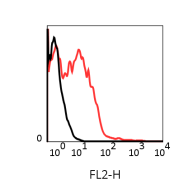

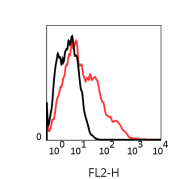

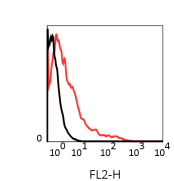

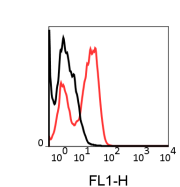


S^--^(OVA)


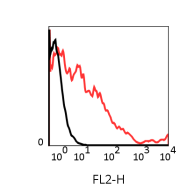

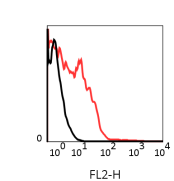

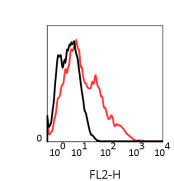

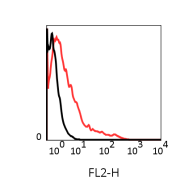

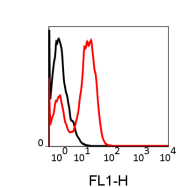


S^-/+^(OVA)


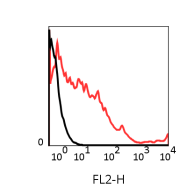

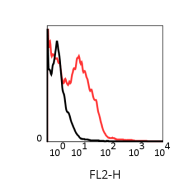

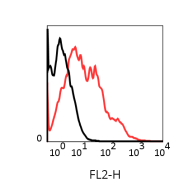

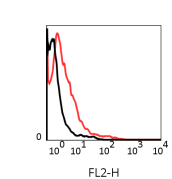

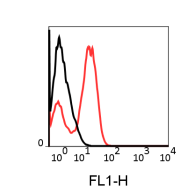


S^-^(OVA)


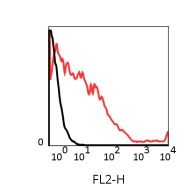

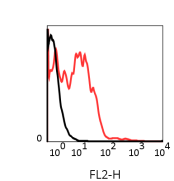

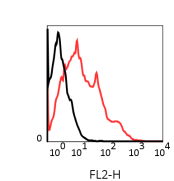

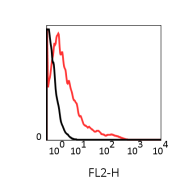


mAb specific against MHC I, MHC II, CD40, CD80 or CD86

Isotype control mAb

**A**

**B**

**Figure S10. Effect of *f*-MWNTs or MWNTs-OVA on BM-DCs phenotypes.** BM-DCs were left untreated or incubated with *f-*MWNTs, OVA or MWNTs-OVA for 24 hr. LPS was used as a positive control. **(A)** Representative histograms for BM-DCs stained with fluorescently labelled antibodies specific to MHC I, MHC II, CD40, CD80, CD86 or their respective isotype controls. The mean florescence intensity (MFI) of the positive cells was determined for 10 x 10^4^ cells with flow cytometry and results were analysed using FLowJo 7.6.5 software. **(B)** The mean fold change in the MFI of each marker compared to naive BM-DC. The mean values of results obtained from two separate experiments ± SD are shown.

**A**

**B**

**Figure S11. Characterization of CD8^+^ T cells and CD4^+^ T cells by immunostaining. (A)** Purity of CD8^+^ T cell isolated from spleen of OT1 Rag^-/-^ using flow cytometryic analysis assessed by CD8 (CD8^+^ T cell marker) expression and absence of CD4 (CD4^+^ T cell marker). **(B)** Purity of CD4^+^ T cell isolated from spleen of OT2 Rag^-/-^ using flow cytometryic analysis assessed by CD4 (CD4^+^ T cell marker) expression and absence of CD8 (CD8^+^ T cell marker).

**A**

**B**

**C**

**Figure S12. Optimization of CD8^+^ T cell: DC ratio, OVA-dose and SIN-dose response *in vitro*. (A)** ^3^H-Thymidine incorporation assay of CD8^+^ T cell co-cultured with BM-DCs, for determination of optimal CD8^+^ T cell: DC number ratio to be used in subsequent studies. BM-DCs stimulated with 1 µg/ml SIN were co-cultured with CD8^+^ T cells at different cell number ratios. CD8^+^ T cells proliferation was determined with ^3^H-thymidine incorporation in CD8^+^ T cells. **(B)** Determination of the *in vitro* CD8^+^ T cell proliferation induced by OVA in a dose-dependent manner. BM-DCs were treated with OVA at concentration ranging from 5 to 40 µg/ml, then co-cultured with CD8^+^ T cells at 1:4 ratio. CD8^+^ T proliferation was measured using ^3^H-thymidine proliferation assay. Results are expressed as mean ± SD (n=3). **(C)** Determination of the CD8^+^ T cells proliferation induced by SIN in a dose-dependent manner. BM-DCs were treated with SIN at concentration ranging from 0.1 to 5 µg/ml, then co-cultured with CD8^+^ T cells at 1:4 ratio. CD8^+^ T proliferation was measured using ^3^H-thymidine proliferation assay. Results are expressed as mean ± SD (n=3).

**A**

**B**

**Figure S13. Assessment of the immune response induced by MWNTs-SIN *in vitro*.** BM-DCs were incubated with SIN or MWNTs-SIN, each at 1 µg/ml SIN for 24 hr. Treated BM-DCs were harvested, irradiated then co-cultured with CD8^+^ T cells at 1:8 ratio for 3 days. **(A)** Assessment of CD8^+^ T cell proliferation with ^3^H-thymidine incorporation assay. **(B)** Measurement of IFN-γ production in the supernatants of CD8^+^ T cells co-cultured with stimulated BM-DCs, by ELISA.

**Figure S14. Assessment of CD8^+^ T cell proliferation induced by SIN in presence or absence of S^-/+^.** BM-DCs were treated with free SIN, SIN and S^-/+^ or S^-/+^(SIN), each at 1 µg/ml SIN, then co-cultured with CD8^+^ T cells at 1:4 ratio. CD8^+^ T cell proliferation was assessed with ^3^H-thymidine incorporation. Experiments were carried out in triplicates. Results are expressed as mean ± SD (n=3).


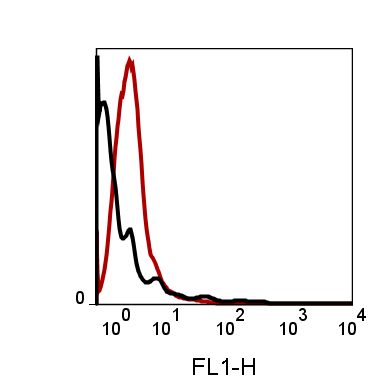

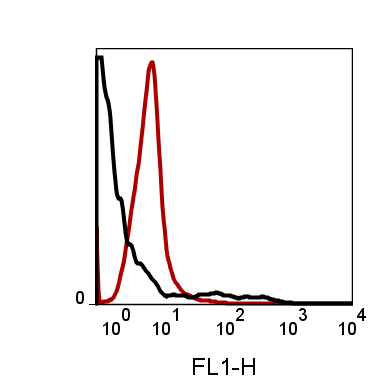

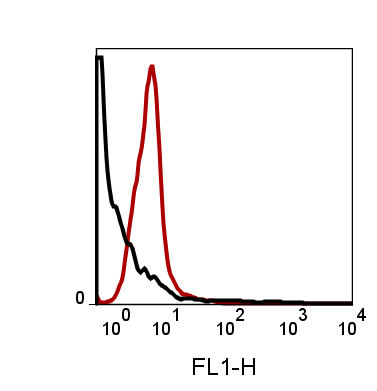

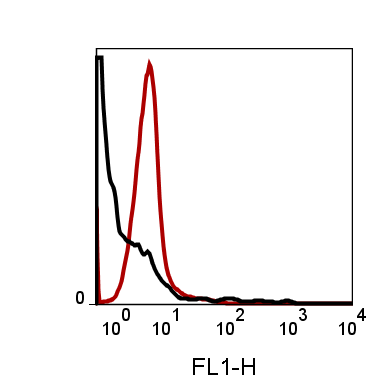

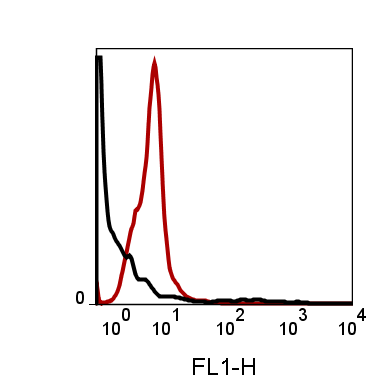

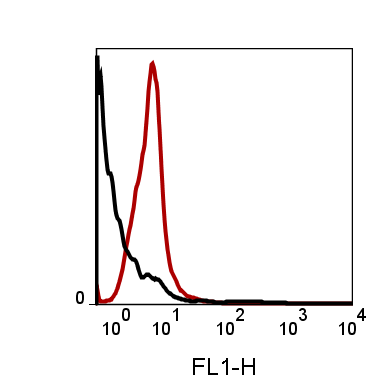

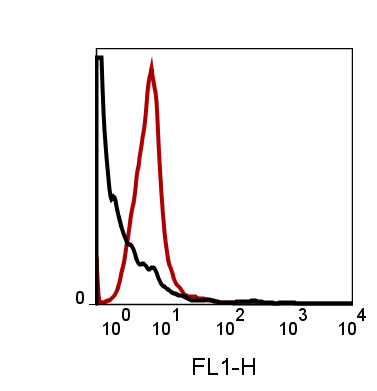

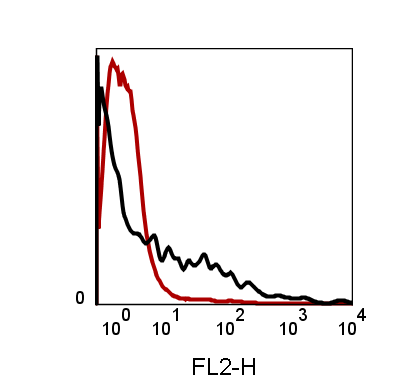

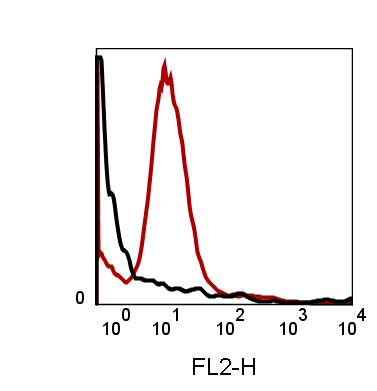

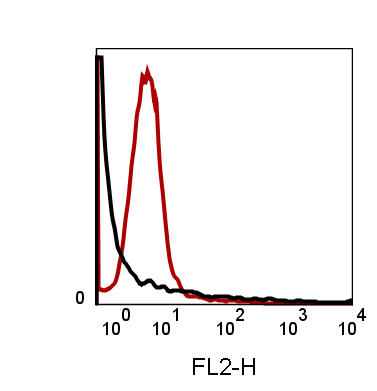

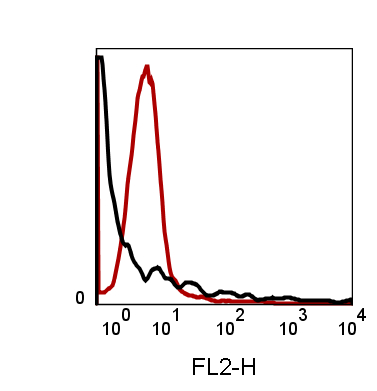

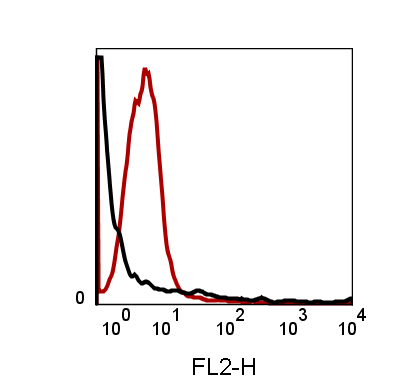

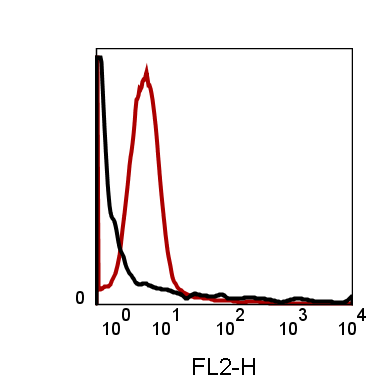

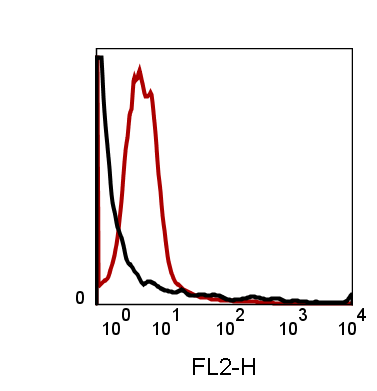

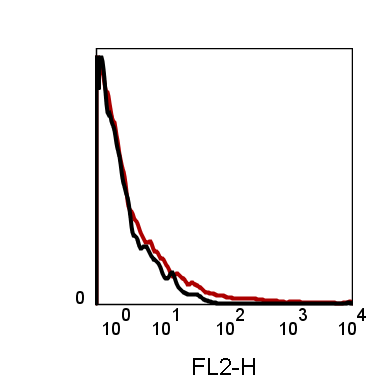

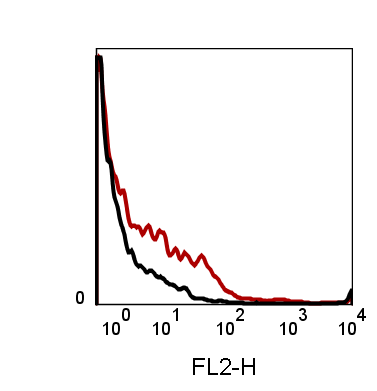

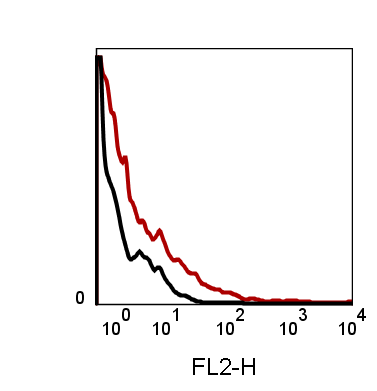

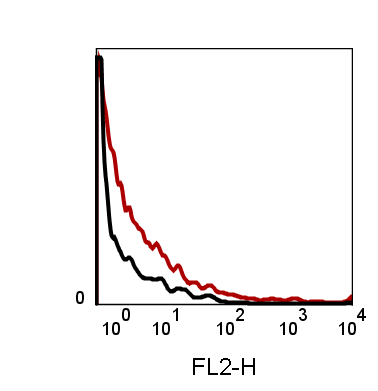

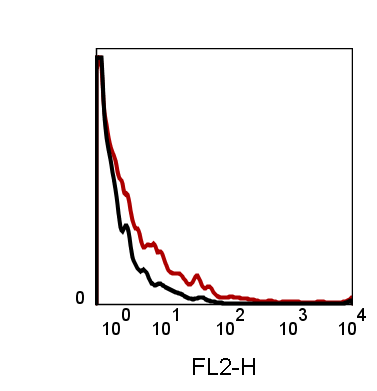

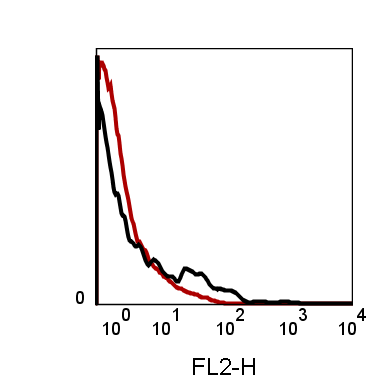

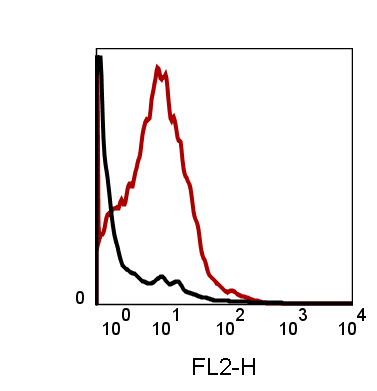

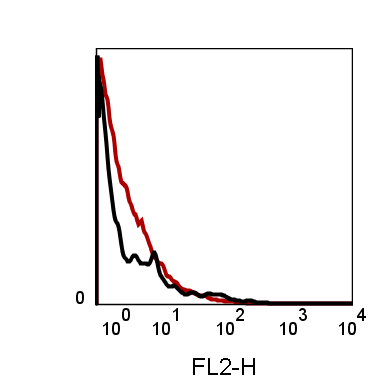

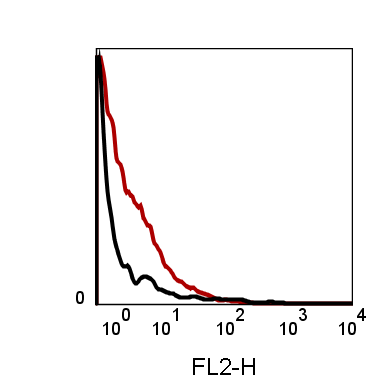

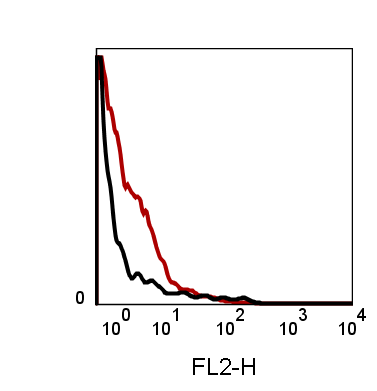

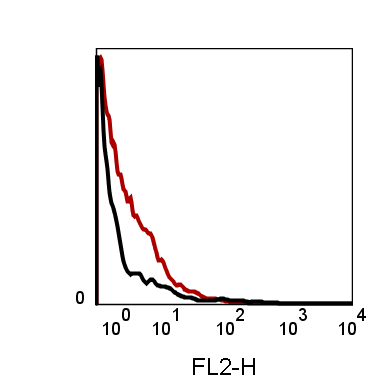

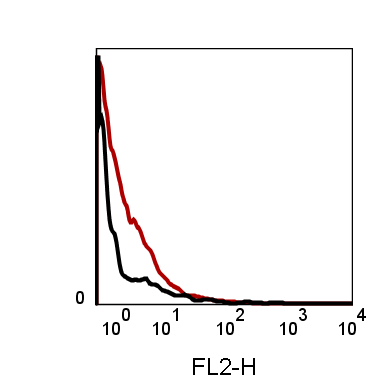

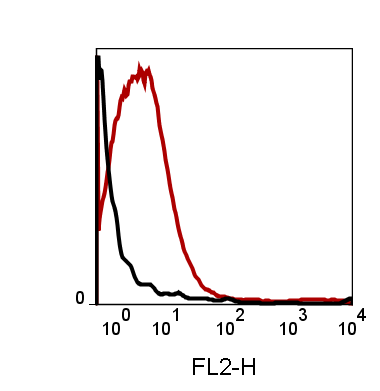

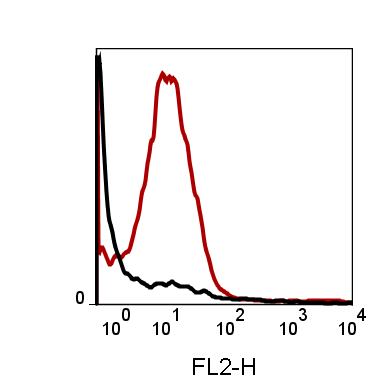

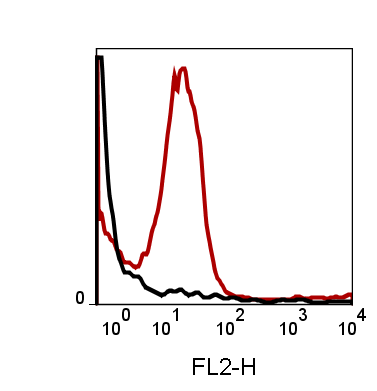

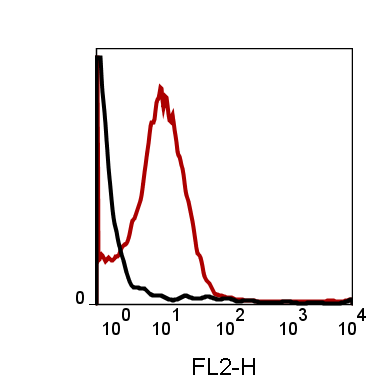

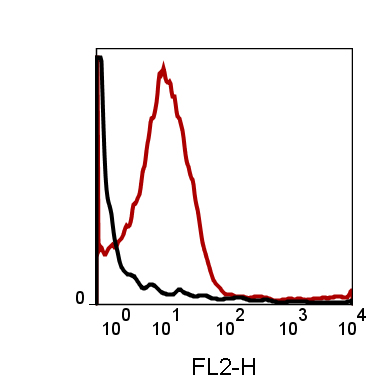

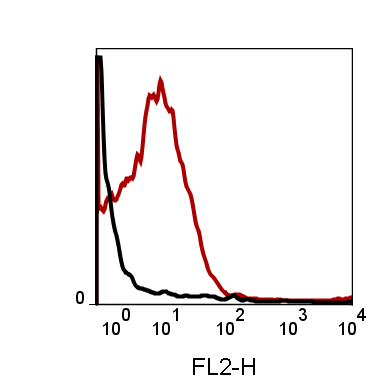

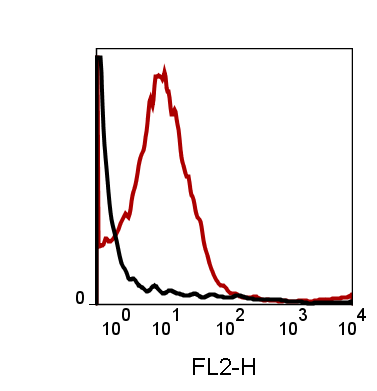

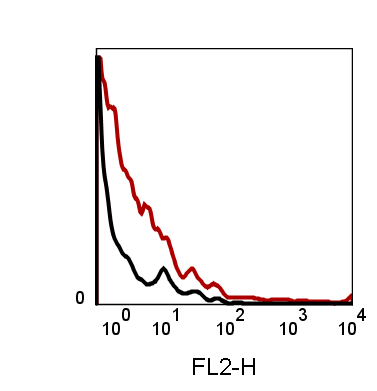

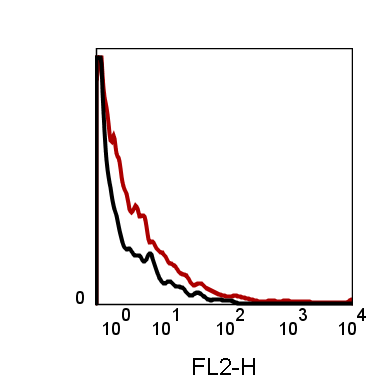


Isotype control mAb

mAb specific against MHC I, MHC II, CD40, CD80 or CD86

Naive

LPS

OVA

L^+^(OVA)

S^--^(OVA)

S^-/+^(OVA)

S^-^(OVA)

MHC I

MHC II

CD40

CD80

CD86

**Figure S15. Effect of MWNTs-OVA on CD11c^+ve^ lymph node cells.** C57BL/6 mice (n=2) were injected via the footpad with OVA or MWNTs-OVA, each contained 50 µg OVA, and the draining popliteal lymph nodes were dissected 24 hrs later. Uninjected or LPS injected mice were used as negative or positive controls, respectively. The lymph node cells were stained with APC anti-CD11c and other fluorescently-labelled antibodies against MHC I, MHC II, CD40, CD80, CD86, or their respective isotype controls and analyzed using flow cytometry. Representative histograms are shown.

**References**

[1] K. Inaba, M. Inaba, N. Romani, H. Aya, M. Deguchi, S. Ikehara, S. Muramatsu, R.M. Steinman, Generation of large numbers of dendritic cells from mouse bone marrow cultures supplemented with granulocyte/macrophage colony-stimulating factor, The Journal of experimental medicine, 176 (1992) 1693-1702.

[2] H. Ali-Boucetta, K.T. Al-Jamal, K. Kostarelos, Cytotoxic assessment of carbon nanotube interaction with cell cultures, Methods Mol Biol, 726 (2011) 299-312.
